# Evaluating machine learning models comprehensively for predicting maximum power from photovoltaic systems

**DOI:** 10.1038/s41598-025-91044-6

**Published:** 2025-03-28

**Authors:** Samir A. Hamad, Mohamed A. Ghalib, Amr Munshi, Majid Alotaibi, Mostafa A. Ebied

**Affiliations:** 1 Process Control Technology Department, Faculty of Technology and Education, Beni-Suef University, Beni Suef, Egypt; 2 Electronics Technology Department, Faculty of Technology and Education, Beni-Suef University, Beni Suef, Egypt; 3 Department of Computer and Network Engineering, College of Computing, Umm Al-Qura University, Makkah, Saudi Arabia

**Keywords:** Maximum power extraction (MPE) technique, Machine-learning, DC–DC converter, Prediction model, Artificial neural network, Electrical and electronic engineering, Energy infrastructure, Mechanical engineering

## Abstract

This paper presents a machine learning (ML) model designed to track the maximum power point of standalone Photovoltaic (PV) systems. Due to the nonlinear nature of power generation in PV systems, influenced by fluctuating weather conditions, managing this nonlinear data effectively remains a challenge. As a result, the use of ML techniques to optimize PV systems at their MPP is highly beneficial. To achieve this, the research explores various ML algorithms, such as Linear Regression (LR), Ridge Regression (RR), Lasso Regression (Lasso R), Bayesian Regression (BR), Decision Tree Regression (DTR), Gradient Boosting Regression (GBR), and Artificial Neural Networks (ANN), to predict the MPP of PV systems. The model utilizes data from the PV unit’s technical specifications, allowing the algorithms to forecast maximum power, current, and voltage based on given irradiance and temperature inputs. Predicted data is also used to determine the boost converter’s duty cycle. The simulation was conducted on a 100 kW solar panel with an open-circuit voltage of 64.2 V and a short-circuit current of 5.96 A. Model performance was evaluated using metrics such as Root Mean Square Error (RMSE), Coefficient of Determination (R^2^), and Mean Absolute Error (MAE). Additionally, the study assessed the correlation and feature importance to evaluate model compatibility and the factors impacting the predictive accuracy of the ML models. Results showed that the DTR algorithm outperformed others like LR, RR, Lasso R, BR, GBR, and ANN in predicting the maximum current (I_m_), voltage (V_m_), and power (P_m_) of the PV system. The DTR model achieved RMSE, MAE, and R^2^ values of 0.006, 0.004, and 0.99999 for I_m_, 0.015, 0.0036, and 0.99999 for V_m_, and 2.36, 0.871, and 0.99999 for P_m_. Factors such as the size of the training dataset, operating conditions of the PV system, model type, and data preprocessing were found to significantly influence prediction accuracy.

## Introduction

In the future, the global demand for energy is anticipated to increase significantly, prompting the need to explore renewable energy sources like geothermal, solar, tidal, and wind power^1^. Among these, solar energy, along with wind power, has seen considerable growth in the global energy sector, driven by decreasing costs and the growing emphasis on reducing greenhouse gas emissions. As a result, there has been a significant rise in the adoption of photovoltaic (PV) systems, particularly in areas with abundant solar radiation, as a strategy to reduce dependence on fossil fuels and protect the environment from pollution^2^.

Various review papers^2–4^ categorize numerous MPPT (Maximum Power Point Tracking) methods based on factors such as sensor requirements, response speed, robustness, memory, and effectiveness. These methods are essential for optimizing system performance by ensuring that the Maximum Power Point (MPP) is achieved under varying temperature and solar radiation conditions. An efficient MPPT controller, typically paired with an inverter or DC-to-DC converter, is crucial in this process. Traditional MPPT techniques, such as Incremental Conductance (IC) and Perturbation and Observation (P&O), are widely used due to their simplicity and ease of implementation^5^. In addition, Karami^6^ introduced other classic algorithms, including Ripple Correlation Control (CC), Open Circuit Voltage (OV), One-Cycle Control (OCC), and Short Circuit Current (SC), which have been effective in scenarios with uniform solar radiation, as demonstrated by Mohapatra^7^. However, a major limitation of these methods is their inability to manage partial shading conditions (PSC), causing the system to lock onto a local MPP, resulting in less efficient energy conversion. To overcome this issue, Ahmed^8^ proposed an enhancement to the P&O approach by using variable step sizes to address challenges like weak convergence, high oscillation, and slow tracking speed. This modification employs larger step sizes when the MPP is far away and reduces them as the system nears the MPP, leading to improved performance. Other variations of modified MPPT methods are also discussed in the literature^2–5^.

Rezk^4^ provides a thorough overview of a distinct class of MPPT control methods based on soft computing strategies. These approaches include artificial neural networks (ANN)^9^, adaptive neuro-fuzzy inference systems (ANFIS)^10,11^, and fuzzy logic control (FLC)^12^. Additionally, various methods leverage evolutionary algorithms such as ant colony optimization (ACO)^13^, the bio-inspired memetic salp swarm algorithm^14^, bee colony algorithm (BCA)^15^, cuckoo search (CS)^16^, bat-inspired optimization (BAT)^17^, and genetic algorithms (GA)^18^. According to Jiang^19^, these techniques, which utilize evolutionary algorithms and soft computing, are particularly effective at addressing nonlinear problems and finding global solutions under partial shading conditions (PSCs). However, they come with two significant limitations: the need for expensive microprocessors to reduce computation time and a reliance on specific knowledge of the PV system. To tackle these challenges, Rizk et al.^4^ proposed a novel approach by integrating Particle Swarm Optimization (PSO) with other algorithms, such as combining GA with PSO^20^ and P&O with PSO, to improve MPPT control^21^.

Extensive research has recently been conducted on reinforcement learning (RL) due to its remarkable ability to learn from historical data and interact with the environment, without the need for complex mathematical models as required by conventional control approaches^22,23^. According to the summary provided by Doltsinis et al.^24^, RL provides a shorter computational time with higher convergence stability compared to meta-heuristic methods, making it a promising solution for optimizing MPPT control. Although there have been limited studies in this area, Q-learning has emerged as the most widely used algorithm. In a study by Wei^25^, MPPT control of a wind power system at different speeds was implemented using a Q-learning algorithm. In^26^, the authors optimized an MPPT controller specifically designed for a tidal energy conversion system. In addition, several studies have explored the application of RL in MPPT control for solar energy conversion systems^24,27,28^. However, these approaches suffer from limitations in the action spaces and states they consider. In^24^, employed five actions and 800 states, resulting in a state-action space of 4000 pairs, while the authors in^27,28^ used only four states. Consequently, systems with larger action spaces and states experience longer computational times. To address this issue, Lai and Phan^29^ proposed a combination of P&O and Q-learning techniques. They employed a Q-learning controller to determine optimal duty cycles for specific control regions based on solar radiation and temperature, and these optimal duty cycles were then used by the P&O controller with a lower step size. Another study by Chou^30^ introduced two RL-based MPPT algorithms, one utilizing a Q table and the other employing a Q network In contrast, other methodologies^31,32^ have addressed the control of MPPT through the use of multiple agents.

However, current research in this field has considered various factors that impact the performance of solar cells, such as temperature and solar radiation, when selecting data features. Some studies have relied on data that captures temperature variations and changing solar radiation throughout the day, rather than using data based on constant temperature and solar radiation. In line with this approach, our study focuses on extracting real-time maximum power from solar cells to develop a machine learning model, with particular attention to the influence of temperature and solar radiation coefficients on the model. This work utilizes data from standalone photovoltaic PV systems to develop prediction models for MPPT using multiple machine learning. The entire machine learning modeling process is explored, including data collection from PV systems, model training, data preprocessing, MPPT modeling, and analysis of influencing factors. The following points summarize the contributions of this study:

- Solar panel data, including irradiance and temperature, were collected from standalone PV systems. MPPT models were established using seven machine learning algorithms, and their effectiveness was assessed utilizing metrics like MAE, R^2^, and RMSE.
- The study takes into account various factors that affect the properties of solar cells when modeling and analyzing their impact.
- The influence of data preprocessing and model selection on the prediction effectiveness of ML algorithms is analyzed.

The structure of the article is explained as follows. In “Methodology” section provides an introduction to evaluation metrics and principles of various machine learning algorithms. In “Case study” section focuses on the standalone photovoltaic (PV) system and covers aspects such as the data collection system, data preprocessing procedures, and the predictive effectiveness of each algorithm. In “Results and discussion” section evaluates machine learning models, considering their predictive performance and interpretability. Finally, “Conclusion” section presents the conclusion of the study.

## Methodology

Machine learning methodologies exhibit formidable self-learning ability and nonlinear fitting, rendering them highly conducive for processing the multi-dimensional data acquired during photovoltaic modeling to achieve optimal energy tracking through the β-MPPT method. Compared to conventional theoretical modeling techniques, their implementation is simpler and they offer the additional advantage of accurately predicting performance under varying operational conditions. As machine learning methods continue to evolve, they enhance the model’s generalization capability and universality.

### Principle of machine learning algorithm

In this section, a prediction model is presented to obtain the maximum current, voltage, and power of a PV system using seven machine learning algorithms: LR, RR, Lasso R, BR, DTR, GBR, and ANN. The principles governing the function of these algorithms will be presented and discussed.

#### Linear regression

The key distinction between logistic and linear regression is their purpose. Linear regression is used to model continuous outcomes, while logistic regression is specifically designed for predicting categorical outcomes with two possible values (e.g. alive/dead, cancer present/absent). Despite this difference, the underlying theories and assumptions are similar for both techniques^33^. However, applying linear regression to dichotomous outcomes can lead to nonsensical predictions^34–36^.

In contrast, logistic regression focuses on estimating the relative likelihood or odds of a particular outcome occurring, rather than trying to predict the exact value. Importantly, the natural logarithm of the odds has a linear relationship over much of its range, allowing the use of techniques developed for linear models.

For more detailed information on logistic regression, the text recommends referring to References^37,38^.

#### Ridge regression

The Ridge Regression (RR) model is used to predict maximum power point tracking (MPPT) values. RR is a technique employed to analyze multiple regression data that exhibits multicollinearity. While least-squares estimates remain unbiased even with multicollinearity, the variances of the estimates can become quite large, potentially leading to discrepancies between the estimates and the actual values. To address this, the RR technique introduces a controlled amount of bias into the regression estimates. This has the effect of reducing the standard errors of the estimates. As a result, it is expected that using RR will improve the reliability and accuracy of the MPPT estimations^39,40^.

#### Lasso regression

Lasso regression, a form of linear regression, incorporates a shrinkage method where data points are pulled towards a central point like the mean^41^. It is designed to encourage the development of simpler, more sparse models with fewer parameters. Lasso resolves this problem by optimizing for a given value of $\lambda$, a non-negative parameter.

1 $$\mathop {\min }\limits_{{\beta_{o} , \beta }} \left( {\frac{1}{2N}\mathop \sum \limits_{i = 1}^{N} \left( {y_{i} - \beta_{o} - x_{i}^{T} \beta } \right)^{2} + \lambda \mathop \sum \limits_{j = 1}^{P} \left| {\beta_{j} } \right|} \right)$$

where $y_{i}$ represents the response at observation $i$, $N$ denotes the number of observations, $x_{i}$ denotes the data, which is a vector of $P$ values at observation $i$, and $\lambda$ stands for a non-negative regularization parameter corresponding to a specific value of Lambda. The parameters are vectors and $\beta_{o}$ are scalars of length $P$. With increasing $\lambda$, the number of non-zero components of $\beta$ decreases.

#### Bayesian regression

Bayesian linear regression uses the Bayesian approach for estimating the parameters within a regression model. This approach involves three main components:

1. *The prior distribution* This represents the initial beliefs about the parameter values before observing any data.
2. *The likelihood distribution* This is the probability of the observed data given the parameter values.
3. *The posterior distribution* This is the updated belief about the parameter values after combining the prior distribution with the likelihood distribution.

In Bayesian linear regression, the parameter estimation process involves deriving the posterior distribution, which is obtained by multiplying the prior distribution by the likelihood^42–44^.

#### Decision trees regression

Decision trees are a type of predictive model that work by iteratively splitting the data into subsets based on different attributes. The construction of a decision tree starts at the root node, where the training data is partitioned according to various attributes. As the tree grows, branches either terminate at leaf nodes when further partitioning becomes impractical, or continue as intermediate nodes.

The decision tree building process continues until the data is optimally classified. The main challenges are selecting which branches to prune and which features to use for splitting the data.

Most decision tree algorithms use a greedy, top-down approach. At each node, the algorithm selects the best feature to split the data into sub-nodes, continuing this process until effective classification is achieved or all attributes have been exhausted.

The information gain is a key metric used to determine the best feature to split on at each node. It is calculated based on the training dataset DD and a given feature AA, the information gain is calculated as follows:

*Step 1*. Compute the empirical entropy of dataset D.

2 $$H\left( D \right) = - \mathop \sum \limits_{k = 1}^{k} \frac{{\left| {C_{k} } \right|}}{\left| D \right|}\log 2\frac{{\left| {C_{k} } \right|}}{\left| D \right|}$$

where $\left| D \right|$ denotes the total number of data sets in D, while $\left| {C_{k} } \right|$ represents the count of samples belonging to the k-th class.

*Step 2*. For data set D, Determine the empirical conditional entropy H(D|A) of feature A.

3 $$H\left( {D|A} \right) = \mathop \sum \limits_{i = 1}^{n} \frac{{\left| {D_{i} } \right|}}{\left| D \right|}H\left( {D_{i} } \right) = - \mathop \sum \limits_{i = 1}^{n} \frac{{\left| {D_{i} } \right|}}{\left| D \right|}\mathop \sum \limits_{k = 1}^{k} \frac{{\left| {C_{ik} } \right|}}{{\left| {D_{i} } \right|}}\log 2\frac{{\left| {C_{ik} } \right|}}{{\left| {D_{i} } \right|}}$$

*Step 3*. Calculate the information gain

4 $$g\left( {D,A} \right) = H\left( D \right) - H\left( {D|A} \right)$$

**Fig. 1:**
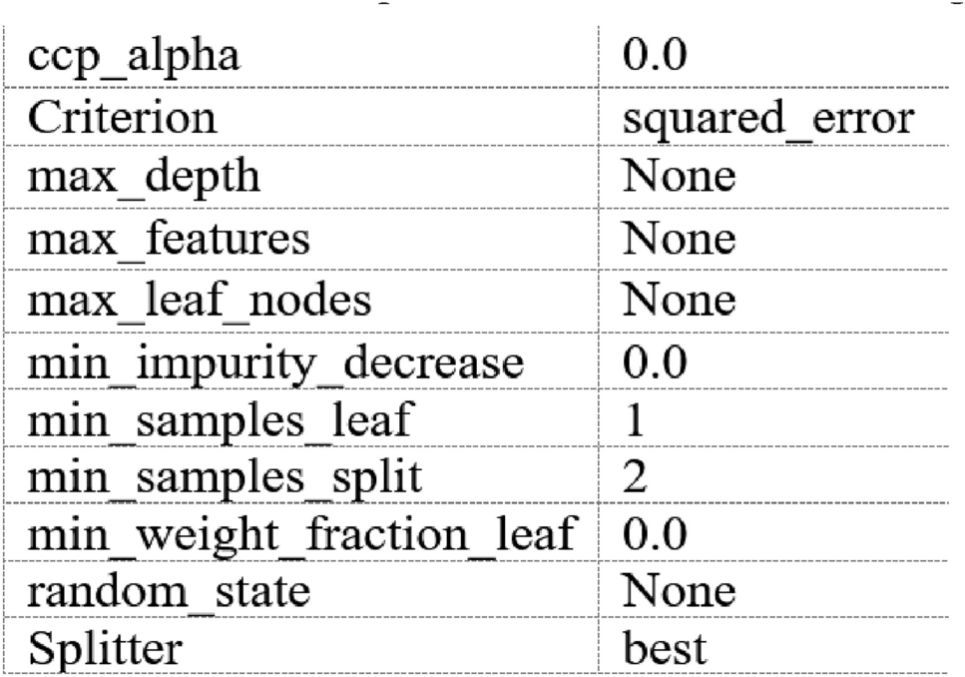
Hyperparameters of the DT method.

In general, a higher information gain indicates a greater improvement in data partitioning efficiency when attributes are used for splitting within the decision tree. Therefore, information gain serves as a criterion for selecting partition attributes in decision trees. The prediction model for MPPT is influenced by the various factors that impact the performance of solar cells, such as temperature and solar radiation. The model parameters are detailed in Fig. 1.

#### Gradient boosting regression

GB is generally known as one of the most realistic and widespread algorithms, representing an improved iteration of boosting trees, the decision tree algorithm. GB employs gradient boosting as its algorithm and utilizes decision trees as its base learner^45,46^. The process of GTB closely follows that of boosting trees in constructing a regression model. However, their divergence lies in the fitting of the loss function: while boosting trees use square loss, GTB employs an approximation method based on the steepest descent. This method revolves around enhancing the regression tree by utilizing the negative gradient value derived from the loss function to estimate the residual error

5 $$F\left( {x,w} \right) = \mathop \sum \limits_{m = 0}^{M} \alpha_{m} h_{m} \left( {x,w_{m} } \right) = \mathop \sum \limits_{m = 0}^{M} f_{m} \left( {x,w_{m} } \right)$$

where $\alpha_{m}$ the weight of each tree, $h_{m}$ the regression tree, $x$ the input sample, $w_{m}$ is the model parameter.

#### Artificial neural network

**Fig. 2:**
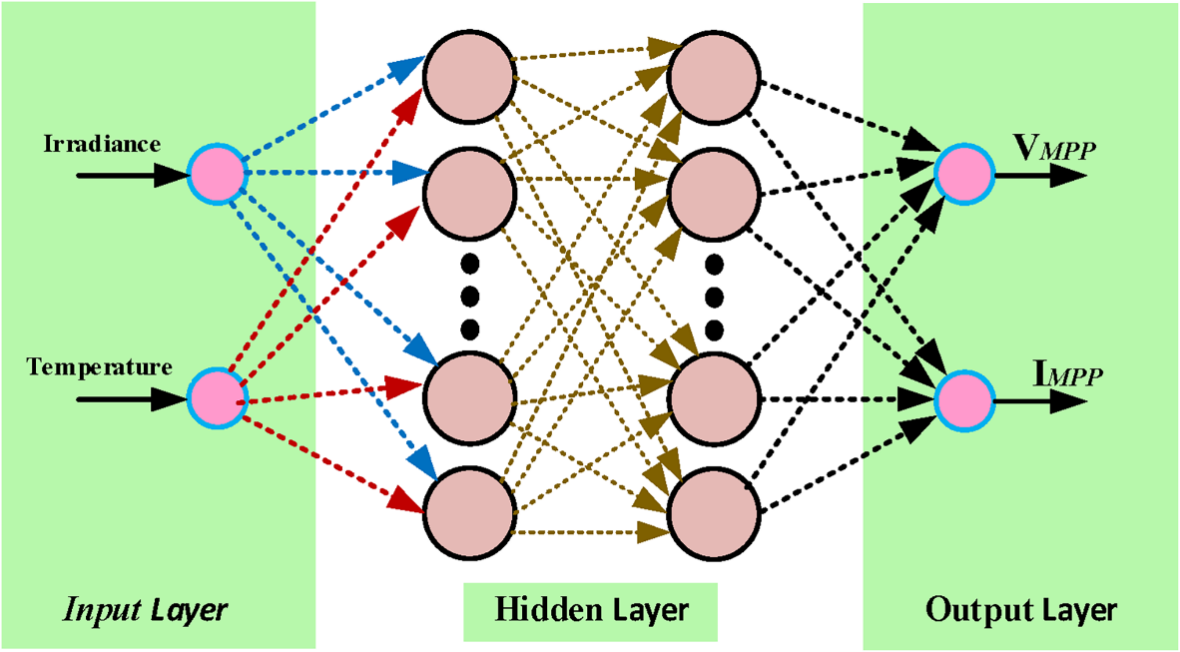
Multilayer perceptron model for MPPT modeling of PV system.

**Fig. 3:**
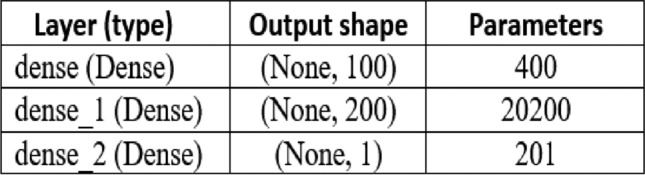
Artificial neural network architecture and parameters.

ANN, or Artificial Neural Network, is a mathematical modeling algorithm that mimics the neural architecture of the brain in terms of information processing^47,48^. It possesses remarkable characteristics, including its capacity to effectively approximate intricate nonlinear functions, adaptability, self-organization, and autonomous learning. Additionally, it demonstrates a considerable level of fault tolerance. The ANN algorithm comprises numerous neurons, each possessing its unique output excitation function. The connections among pairs of neurons are represented as weights, indicating the ANN’s memory capacity. Neurons within an ANN are categorized as output, hidden, or input neurons.

6 $$t = f\left( {W_{i}^{T} A_{i} + b} \right)$$

where, the output or predicted data is $t$, the input components, labeled as $A_{1}$, $A_{2}$ …, $A_{n}$, contribute to this computation. Corresponding to each component, there exist weights denoted as $W_{1}^{T}$, $W_{2}^{T}$ …, $W_{n}^{T}$, along with a bias term denoted as $b.$ The activation function $f$, typically chosen from popular options such as sigmoid, relu, or tanh, determines the output of the individual neurons in the network. The ANN model utilized in this research comprises three layers: an input layer consisting of independent variables, an output layer comprising dependent variables, and a hidden layer. The Python environment is where the current ANN model, based on theoretical and multi-layer cognitive models, has been implemented. A specific learning algorithm is employed to adjust the network weights for training purposes, with the dense backpropagation algorithm utilized in this study. In order to improve the effectiveness of artificial neural networks, the relu activation function is utilized. The architecture of the model consists of three layers: an input layer, a hidden layer, and an output layer. Figure 2 illustrates the multilayer perceptron model of the PV system. The intelligent structure and training parameters of the artificial neural network are provided in Fig. 3.

### Metrics for evaluating the ML model

Equation (7) defines the Mean Absolute Error (MAE), used alongside R^2^ and RMSE to evaluate the predictive performance of seven machine learning algorithms for estimating Maximum Power Point Tracking (MPPT). These metrics are vital for assessing model reliability and accuracy, aiding in their improvement.

7 $$MAE = \frac{1}{m}\mathop \sum \limits_{i = 1}^{m} \left| {\left( {y_{i} - \widehat{{y_{i} }}} \right)} \right|$$

In this context, $y_{i}$ represents the actual data, while $\widehat{{y_{i} }}$ represents the predicted data. When evaluating a model, if the predicted values (which can range from (0, + ∞)) perfectly match the true values, the Mean Absolute Error (MAE) would be 0, indicating an ideal model.

On the other hand, as the model’s predictions deviate more from the actual values, the MAE increases. Therefore, a lower MAE value indicates that the prediction model is more accurate, while a higher MAE suggests that the model’s predictions are less accurate.

8 $$R^{2} = 1 - \frac{{\mathop \sum \nolimits_{i} \left( {\widehat{{y_{i} }} - y_{i} } \right) }}{{\mathop \sum \nolimits_{i} \left( {\overline{{y_{i} }} - y_{i} } \right) }}$$

The R^2^ statistic provides comprehensive insights without the interpretability challenges often associated with other metrics. It plays a crucial role in evaluating regression models^45,49^.

R^2^ values that approach 1 indicate higher predictive accuracy. By comparing R^2^ values across different models, researchers can determine which model performs best and identify the factors that contribute to its effectiveness. This allows for focused efforts to improve model performance.

Generally, a higher R^2^ value suggests that the model fits the data better, meaning it explains a larger proportion of the variance in the dependent variable. RMSE, as depicted in Eq. (9).

9 $$RMSE = \sqrt {\frac{1}{N}\mathop \sum \limits_{i = 1}^{N} \left( {y_{i} - \widehat{{y_{i} }}} \right)^{2} }$$

In evaluating performance, *y*_*i*_ is the predicted score and $\widehat{{y_{i} }}$ is the actual score in the test dataset. *N* is the total number of prediction pairs. Lower RMSE values indicate a better model fit. When comparing regression models, choose the one with the smallest RMSE for superior predictive accuracy.

The Normalized Root Mean Squared Error (NRMSE) is a valuable metric for evaluating the performance of predictive models, particularly when comparing models across different scales. The NRMSE is calculated using the following equation:

10 $${\text{NRMSE}} = \frac{{\sqrt {\frac{1}{{\text{N}}}\mathop \sum \nolimits_{{{\text{i}} = 1}}^{{\text{N}}} \left( {{\text{y}}_{{\text{i}}} - \widehat{{{\text{y}}_{{\text{i}}} }}} \right)^{2} } }}{{{\text{y}}_{max} - {\text{y}}_{{{\text{min}}}} }}$$

where N is the number of data points, ${\text{y}}_{{\text{i}}}$ represents the actual values, $\widehat{{{\text{y}}_{{\text{i}}} }}$ represents the predicted values, ${\text{y}}_{max}$ is the maximum value of the actual data and ${\text{y}}_{{{\text{min}}}}$ is the minimum value of the actual data.

The NRMSE provides a normalized measure of the prediction error, making it easier to interpret and compare across different datasets with varying scales. By dividing the RMSE by the range of the actual values $\left( {{\text{y}}_{max} - {\text{y}}_{{{\text{min}}}} } \right)$, the NRMSE accounts for the scale of the target variable, allowing for a more balanced and meaningful comparison of model performance. This metric is particularly useful in fields where the target variable can vary significantly in magnitude, ensuring that the error measure is relative to the data’s inherent scale.

## Case study

### Standalone photovoltaic (PV) system

**Fig. 4:**
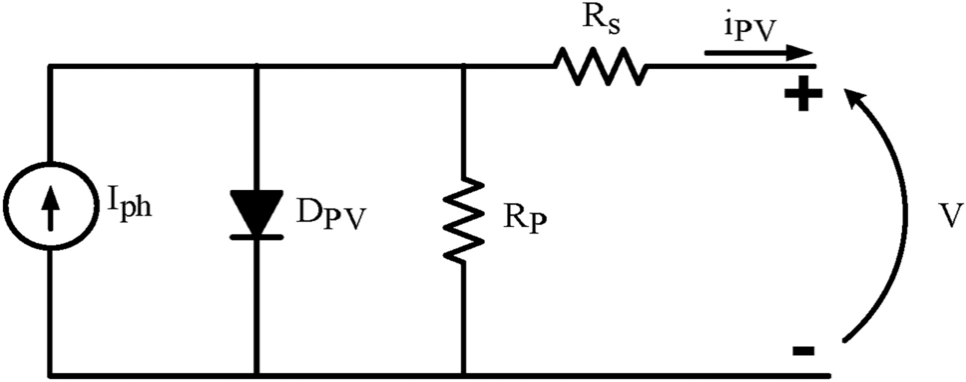
The equivalent PV circuit model.

Photovoltaic (PV) cells use the photoelectric effect to transform solar energy into direct current (DC) electrical energy. To increase both voltage and current output, these cells are connected in series and parallel arrangements to form PV arrays. A detailed circuit model representing a solar PV cell is shown in Fig. 4^50^. This model helps illustrate the electrical characteristics and behavior of a single PV cell within the larger array system^51,52^.

11 $$I_{pv} = I_{ph} - I_{r} \left[ {\exp \left( {\frac{{q\left( {V + R_{s} I_{pv} } \right)}}{\eta kT}} \right) - 1} \right] - \frac{{V + R_{s} I_{pv} }}{{R_{p} }}$$

12 $$I_{{ph}} = \left\lceil {I_{{sc}} + \alpha \left( {T - T_{{ref}} } \right)} \right\rceil \frac{G}{{1000}}$$

13 $$I_{r} = I_{{rr}} \left( {\frac{T}{{T_{{ref}} }}} \right)^{3} e^{{\left\lceil {\left( {qE_{g} /\eta k} \right)\left( {\left( {1/T_{{ref}} } \right) - \left( {1/T} \right)} \right)} \right\rceil }}$$

14 $$I_{rr} { } = \frac{{I_{sc} { } - \left( {V_{oc} /R_{p} } \right)}}{{\left[ {\exp \left[ {\frac{{qV_{oc} }}{{\eta kT_{ref} }}} \right]} \right] - 1}}{ }$$

15 $$V_{oc} \left( T \right) = V_{oc} \left( {T_{ref} } \right) + \beta \left( {T - T_{ref} } \right)$$

**Fig. 5:**
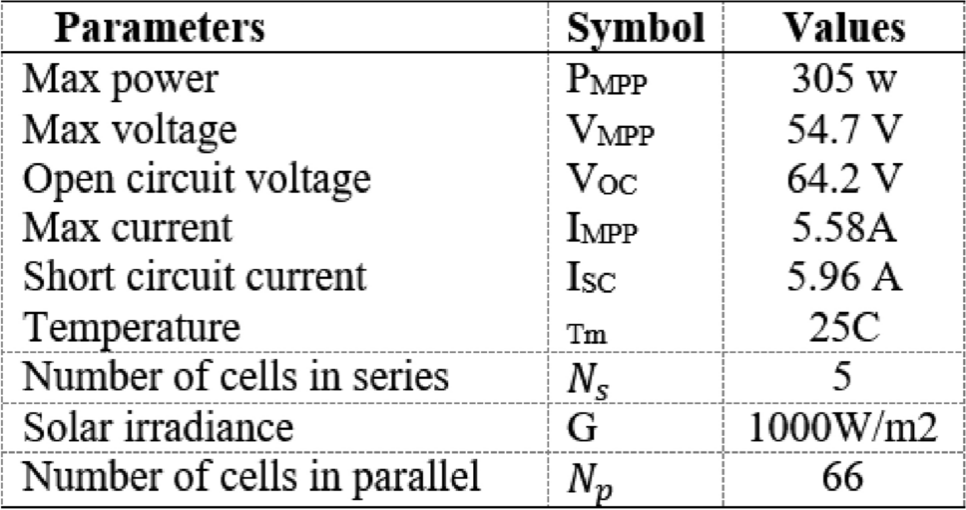
Electrical characteristics of the Sun power solar cells.

**Fig. 6:**
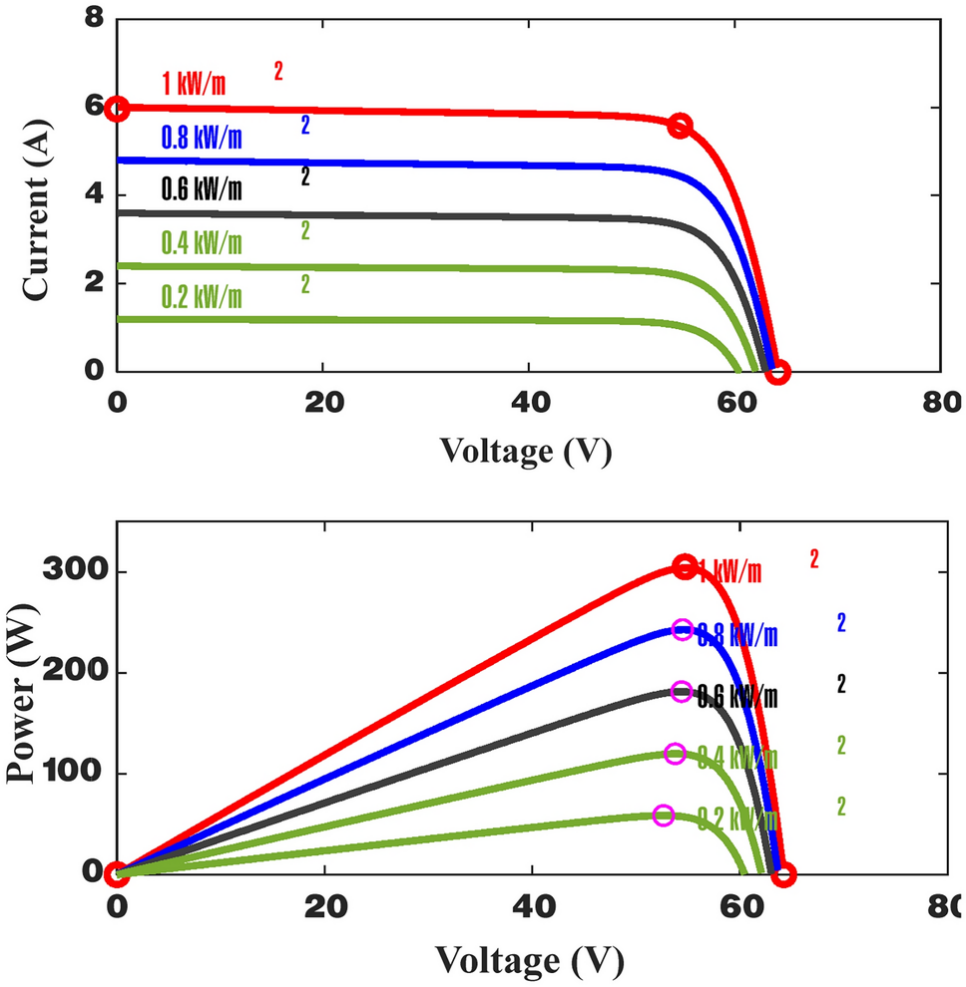
Characteristics of variable irradiance PV panels at 25 °C.

**Fig. 7:**
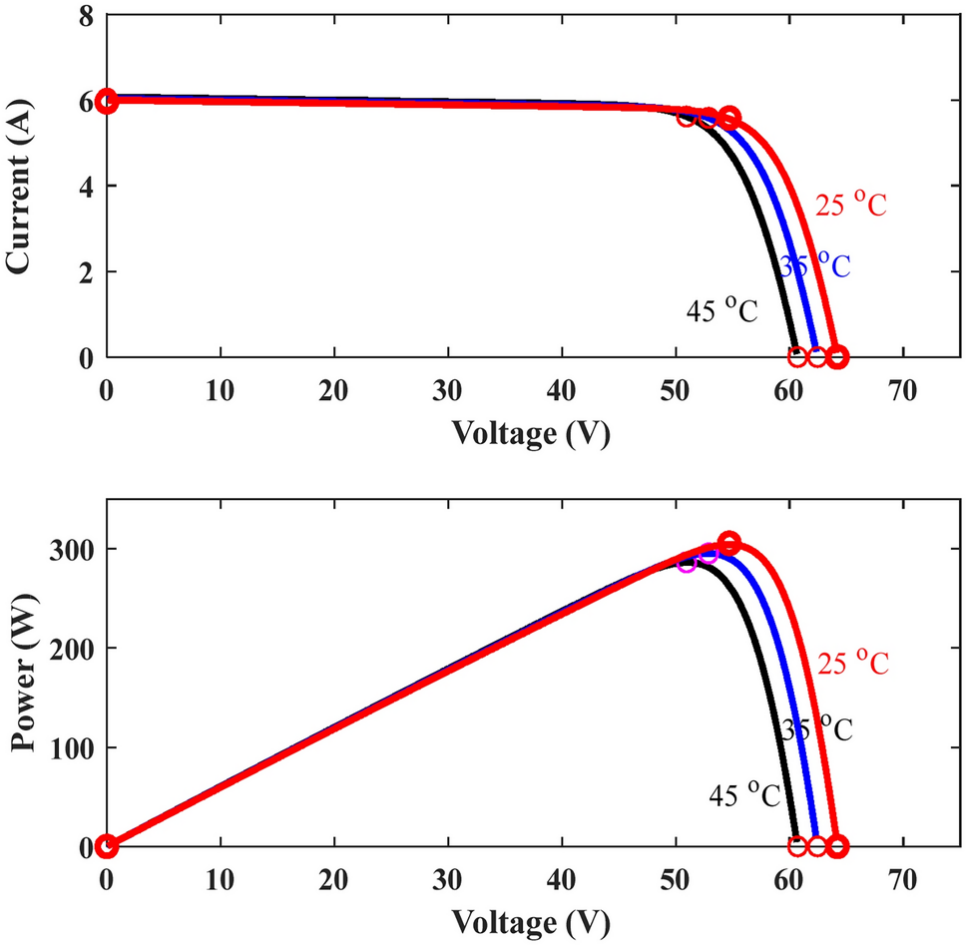
Characteristics of variable temperatures PV panels at1000 W/m^2^.

The proposed methodology comprises two primary steps. Initially, multiple machine learning (ML) models are constructed using data derived from the parameters of the PV panel. These models are then utilized for MPPT. The first step involves considering irradiance (I_r_) and temperature (T) as functions of the P_m_ and the corresponding V_m_ at the MPP. The created models are subsequently used to forecast P_m_ and V_m_ based on specified I_r_ and T values. Following this, the required duty cycle (D) is calculated using the expected values to obtain the MPP of the PV. Figures 5, 6 and 7 show the PV specifications for all irradiance and temperature variations.

#### MPPT with boost converter

**Fig. 8:**
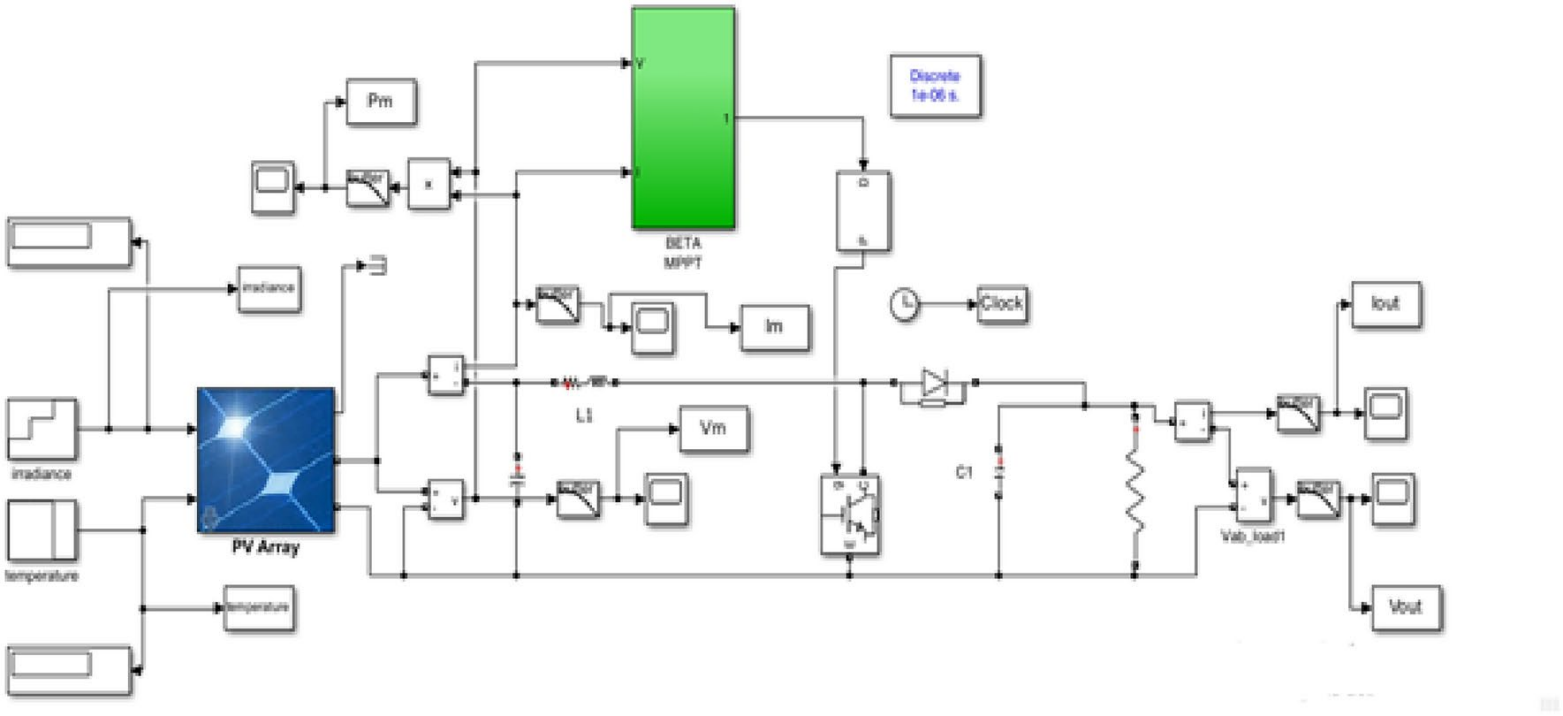
Model of a PV connected to a boost converter.

A DC–DC boost converter is illustrated in Fig. 8, which is powered by a photovoltaic (PV) panel. The converter’s transistor duty ratio (D) controls power transfer from the panel to the load, with an inductor (L) raising PV voltage to the desired output level. Ripple in output voltages is minimized by output (C_o_) and input (C_i_) capacitors. Boost inductor current increases linearly during switch-on and diode-off periods, releasing stored energy through the diode when the switch is off. The capacitive filter ensures smooth switching action while providing a constant DC voltage to the load.

Ayop et al.^53^ introduced a method for determining the range of load resistance, specifically its upper and lower boundaries.

Rashid^54^ offered a technique for designing boost converters. In this context, two key equations are provided:

- Equation (16) defines the inductance necessary for a boost converter.
- Equation (17) specifies the required capacitance for the converter.

These equations and methods are crucial for the proper design and functioning of boost converters in various applications.

16 $$L = \frac{{V_{ip} \times \left( {V_{op} - V_{ip} } \right)}}{{f_{sw} \times \Delta I \times V_{op} }}$$

17 $$C = \frac{{I_{op} \times \left( {V_{op} - V_{ip} } \right)}}{{f_{sw} \times \Delta V \times V_{op} }}$$

The ML model is trained to predict the Pm and corresponding V_m_ of the PV module based on the features I_r_ and T. Using the forecasted P_m_ and V_m_ values from Eq. (18), the resistance ${\text{R}}_{{{\text{mp}}}}$ at the MPP is calculated. By adjusting the converter’s duty cycle (D), as shown in Eq. (19), the load resistance $R_{o}$ and ${\text{R}}_{{{\text{mp}}}}$ determine the converter’s behavior.

18 $${\text{R}}_{{{\text{mp}}}} = \frac{{V_{m} }}{{I_{m} }}$$

19 $$D = 1 - \sqrt {\frac{{R_{mp} }}{{R_{o} }}}$$

#### β-MPPT method

The β-MPPT approach introduces the concept of monitoring an intermediary variable labeled β instead of tracking changes in power, as described by Eqs. (20) and (21).

20 $${\upbeta } = {\text{ln}}\left( {\frac{{{\text{i}}_{{{\text{PV}}}} }}{{{\text{v}}_{{{\text{PV}}}} }}} \right) - {\text{C}} \times {\text{v}}_{{{\text{PV}}}}$$

21 $${\text{C}} = \frac{{\text{q}}}{{{\text{NnKT}}}}$$

where $T$ is the temperature, $K$ is the Boltzmann constant, the diode constant is $C$, ${\text{V}}_{{{\text{PV}}}}$, is the voltage, and, ${\text{i}}_{{{\text{PV}}}}$ is the photovoltaic module current, $q$ is the electron charge. N denotes to the number of photovoltaic cells in the module.

**Fig. 9:**
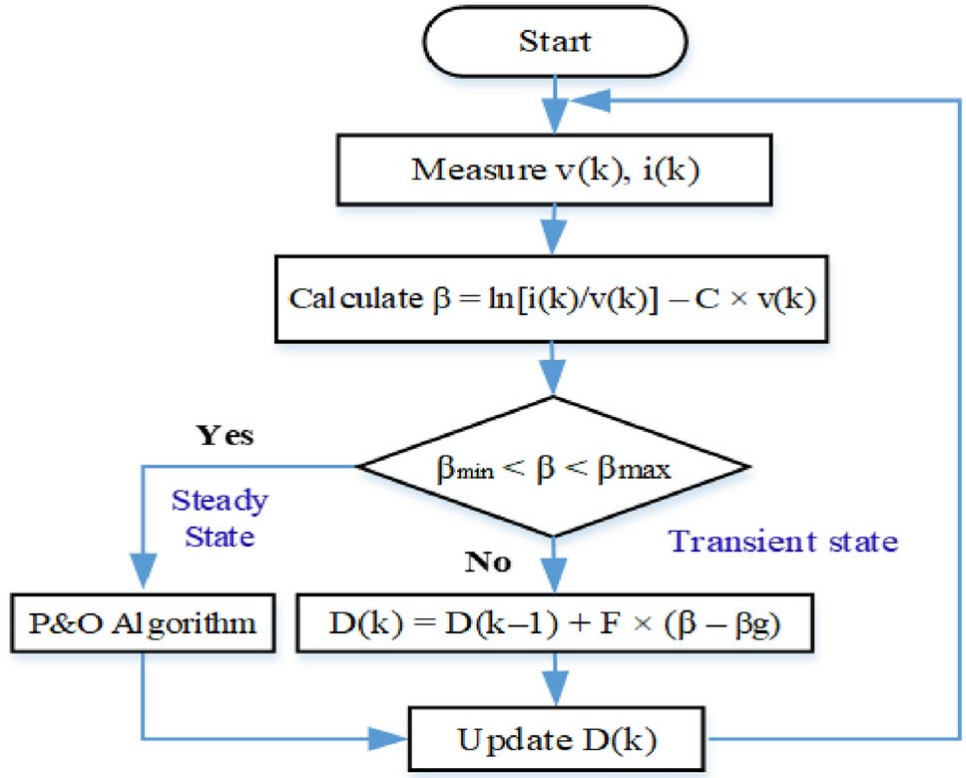
Flowchart of the β-MPPT technique.

This method employs a two-phase approach: variable steps during the transient stage and fixed steps in the steady-state phase^55^. The process is visually represented in the flowchart shown in Fig. 9.

The initial step involves monitoring the current and voltage before continuously calculating β values. The next action depends on the calculated β value:

1. If β falls within the range (βmin, βmax), the Beta technique transitions to its steady-state phase.
2. If β is outside this range, the system enters the transient stage, where the Perturb and Observe (P&O) strategy is implemented.

During the transient stage, which is temporary, the variable step size ΔD is computed using a guiding parameter β_g_. This calculation is represented by Eq. (22).

This approach allows for adaptive control, balancing rapid response during transient conditions with stability during steady-state operation.

22 $$\Delta D = F \times \left( {\beta - \beta_{g} } \right)$$

where F represents the scaling factor.

The irradiance and temperature variables have an impact on the range of the β parameter. The values of F = 0.01, βmin = 15.45, and βmax = 19.02 were set in this study. The average value of βmin and βmax, β_g_ = 17.24, was employed.

Particle swarm optimization (PSO) in combination with the beta MPPT controller to improve PV system performance.

The scaling factor (F) plays a crucial role in dynamically adjusting the step size (∆D) in the transient state of the Beta MPPT strategy. An optimal value of (F) ensures:

- Fast convergence to the Maximum PowerPoint (MPP).
- Reduced steady-state oscillations.
- High efficiency under varying irradiance and temperature.

Since F significantly affects the MPPT performance but lacks a direct analytical solution, a meta-heuristic optimization algorithm can be employed to determine its optimal value.

PSO provides a robust control strategy under multiple load conditions and with different system inputs. This increases the adaptability and robustness of our control system. This not only improves the performance, but also ensures that the Beta MPPT operates optimally under different conditions.

#### Meta-heuristic technique

The objective function must optimize (F) to maximize power extraction efficiency (η) while minimizing steady-state oscillations (SSO) and convergence time (CT).

23 $${\text{J}}\left( {\text{F}} \right) = {\text{W}}_{1} \times \left( {{1} - \, \eta } \right) + {\text{ W}}_{2} \times {\text{SSO }} + {\text{ W}}_{3} \times {\text{CT}}$$

where:

- MPPT Efficiency (η = $${\text{P}}_{{{\text{MPPT}}}} /{\text{P}}_{{{\text{ideal}}}}$$)
- SSO = Root Mean Square of Power Oscillations.
- CT = Time taken to reach 98% of ${\text{P}}_{{{\text{ideal}}}}$.
- ${\text{W}}_{1}$, ${\text{W}}_{2}$, ${\text{W}}_{3}$ are weight factors.

The goal is to minimize J(F).

**Fig. 10:**
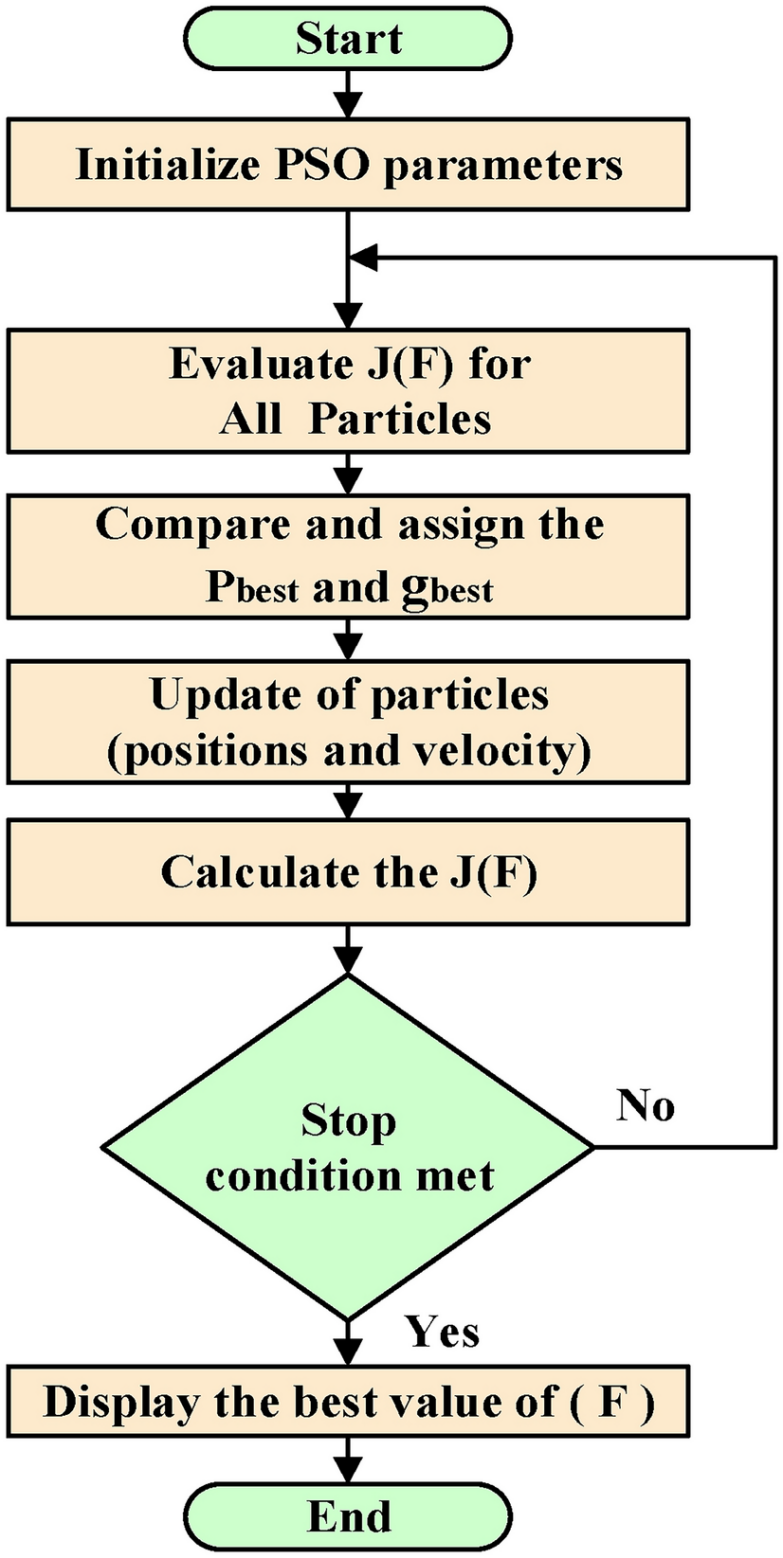
Flow chart of the PSO.

**Fig. 11:**
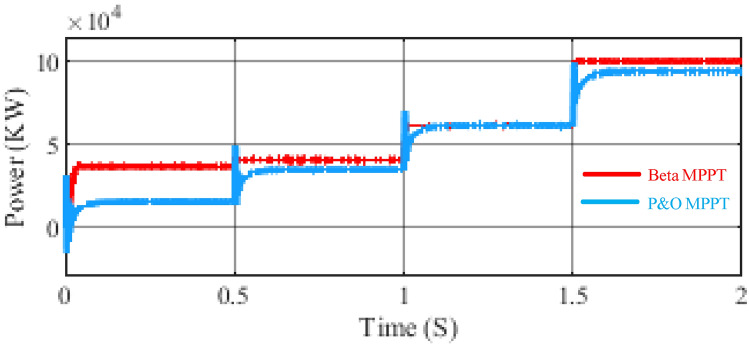
Comparison of power output between traditional P&O and beta MPPT.

Figures 10 and 11 presents a detailed comparison between the traditional Perturb and Observe (P&O) Maximum Power Point Tracking (MPPT) method and the beta MPPT method in terms of power output efficiency. It is evident from the figure that the beta MPPT method demonstrates superior accuracy in tracking the maximum power point (MPP) of the photovoltaic (PV) solar cell under varying environmental conditions. Specifically, the beta MPPT method consistently achieves more precise power extraction across a wide range of solar irradiance levels and temperature variations. In contrast, the traditional P&O MPPT method exhibits noticeable deviations from the optimal power point, particularly under rapidly changing environmental conditions. These findings highlight the enhanced adaptability and efficiency of the beta MPPT method, making it a more reliable for maximizing power generation in dynamic and unpredictable solar energy environments.

### Dataset collection

Data were collected from MATLAB to determine the maximum power of solar cells under different operating conditions. Initially, the parameters of interest such as current (I), voltage (V), temperature (T), solar radiation, and power (P) were determined. A solar panel with a maximum power specification of 100 kW, a short-circuit current of 5.96 A, and an open-circuit voltage of 64.2 V was utilized. A simulation was conducted in MATLAB employing B-MPPT algorithms to track the maximum power point (MPP) under varying conditions like temperature fluctuations and changing solar radiation levels to encompass a range of operational scenarios. Relevant data points including current, voltage, power, and corresponding conditions were extracted from the MATLAB simulation. Subsequently, this data was organized for analysis and model training to enhance the understanding of the behavior of solar cells under various operating conditions.

### Data preprocessing

**Fig. 12:**
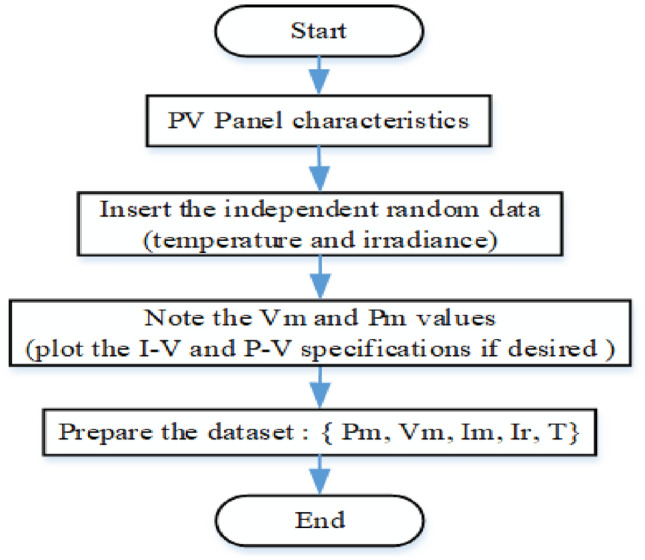
Diagram illustrating the methodological steps of data collection.

Figure 12 illustrates a systematic approach to gathering and organizing data for analyzing PV panel performance under various environmental conditions. This analysis is vital for predicting and optimizing solar panel efficiency. The dataset necessary for testing and training the model comprises Irradiance (Ir), Temperature (T), Power (P), Current (I), and Voltage (V).

In this study, a solar panel with a maximum power capacity of 100 kW, a short-circuit current of 5.96 A, and an open-circuit voltage of 64.2 V was utilized. The dataset required for training and testing the model included solar irradiance (Ir), temperature (T), power (P), voltage (V), and current (I). These parameters were generated through simulations conducted in MATLAB using the B-MPPT method under varying operating conditions, such as changes in temperature and solar irradiance. The simulation model, depicted in Fig. 5, was executed to extract the necessary data. The values of Ir, P, V, and I were transferred to the MATLAB workspace and saved as variables, which were subsequently exported in Excel format for further processing. This structured dataset was then organized and analyzed to support the development and training of the model. The goal of this process was to enhance the understanding of solar cell behavior under dynamic operating conditions and improve the efficiency of the MPPT method.

During the data analysis and cleaning phase, instances of duplicated data points within the dataset were discovered. This duplication occurred because data in the output power was repeated due to slight deviations from the required value, as there were minor increases or decreases in power to search for the desired value. Approximately 1% of the dataset contains duplicate entries, with variables like maximum power being notably affected. In this study, tools such as Python’s Pandas library were utilized to address this issue. The duplicated () function was employed to identify rows with identical values across columns, specifying relevant columns using the subset parameter when necessary. The drop_duplicates() method was then used to eliminate duplicate entries from the dataset. By default, this method retains the first occurrence and removes subsequent duplicates, though its behavior can be customized, such as keeping the last occurrence, by specifying the keep argument. In total, 876,012 data points were collected for this work, and after data preprocessing, 850,793 data points were available for modeling.

### Prediction performance

**Fig. 13:**
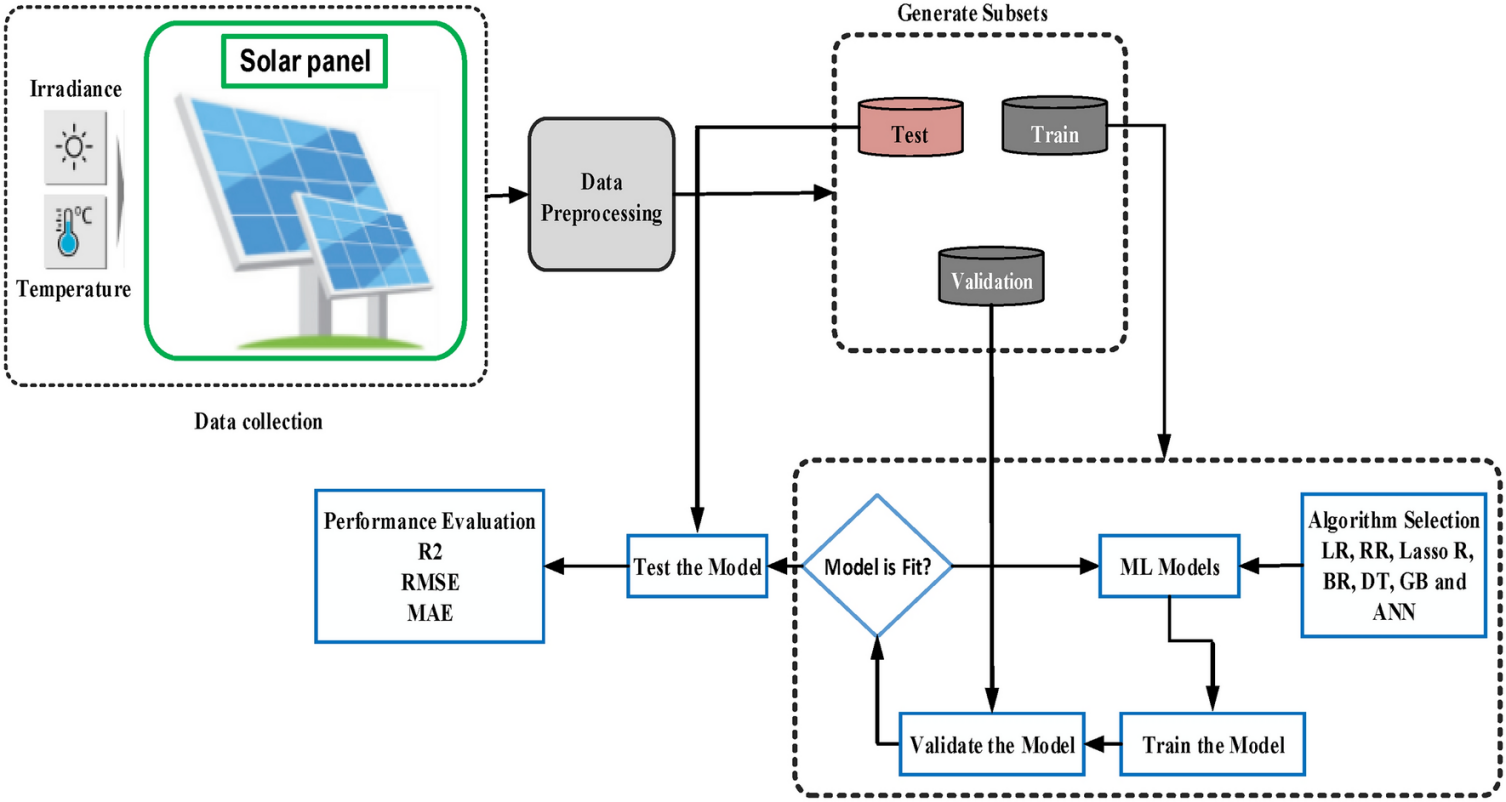
Flow diagram of the steps used in the ML models and evaluation process.

To evaluate the performance of the machine learning model, the dataset was divided randomly into training (80%) and validation/testing (20%) sets. Stratified sampling was utilized to maintain the distribution of key demographic variables in both sets. Using Python software, a model was developed to predict MPPT tracking. The software operates in a Python 3.9.13 [MSC V. 1916 64bit (AMD 64)] environment, running on a 64-bit Windows 10 operating system. The CPU used is an i5-8300H 2.30GHz, Intel(R) Core(TM) processor. The steps of this process are shown in Fig. 13.

**Fig. 14:**
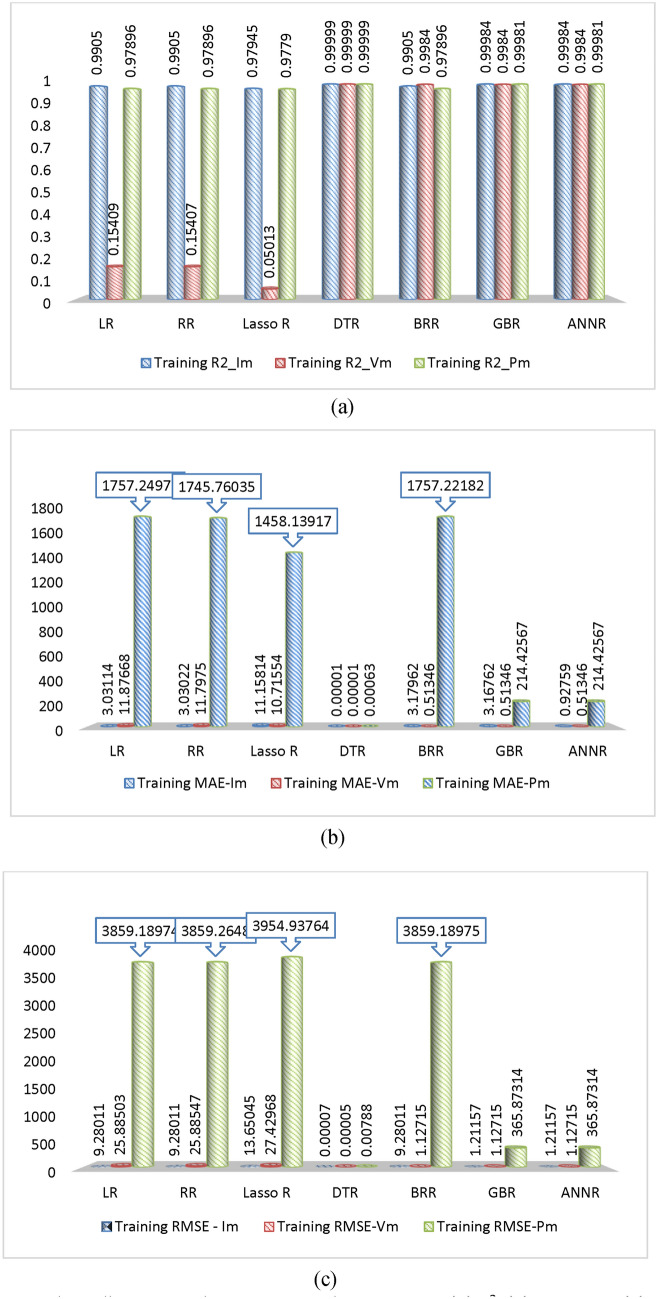
Bar chart illustrating the training evaluation using (**a**) R^2^, (**b**) MAE, and (**c**) RMSE as evaluation metrics for all ML models.

In this study, we conducted a comparative analysis of seven machine learning algorithms: LR, RR, Lasso R, BR, DTR, GBR, and ANN. Figure 9 illustrates the performance of each algorithm during training. As shown in Fig. 14a, all algorithms demonstrate high training effectiveness, with R^2^ values exceeding 0.97 for predicting I_m_, V_m_, and P_m_. DTR notably delivers the best prediction performance for maximum current, voltage, and power, achieving an R^2^ of 0.99999. Training times across algorithms are fairly consistent, with ANN taking the longest at 1.059 s. Figure 14b highlights the strong performance of each algorithm in terms of MAE, with values below 0.00002, 0.0002, and 0.00073 for predicting I_m_, V_m_, and P_m_, respectively. Once again, DTR stands out with MAE values of 0.00001, 0.00001, and 0.0063 for these predictions. Similarly, Fig. 14c shows that all algorithms perform well, with RMSE values under 0.00085, 0.00006, and 0.00891 for predicting I_m_, V_m_, and P_m_, respectively. DTR continues to demonstrate the highest prediction accuracy, achieving RMSE values of 0.000075, 0.00005, and 0.00788 for I_m_, V_m_, and P_m_, respectively. As a result, DTR emerges as the top-performing algorithm for predicting maximum current, voltage, and power. It is important to note that this study specifically focuses on solar cells and uses collected data samples to determine maximum power output. Variations in data sources and characteristics may influence the accuracy and performance of the models.

## Results and discussion

### Evaluating the interpretability of ML models

Although DTR, BRR, GBR, and ANN contribute to the explanatory power of the model, the interpretability of these models remains a significant concern in modeling. Machine learning models, in general, are often regarded as having limited interpretability. Therefore, it is necessary to perform correlation and significance analysis to address this issue.

Decision Tree Regression (DTR) and Gradient Boosting models in PV system applications face several limitations that require careful consideration. These limitations have been effectively overcome through several strategic approaches which include the following:

DTR overfitting, several key measures have been implemented. These included utilizing pruning techniques, employing cross-validation for model validation, and setting appropriate maximum depth parameters. Additionally, regular validation of model performance with new data and the use of ensemble methods significantly improved generalization capabilities.

The computational costs associated with Gradient Boosting have been managed through several optimization strategies. This involved optimizing hyperparameters for efficiency, utilizing lightweight implementations, and considering hardware acceleration options. Furthermore, implementing batch processing and scheduling model updates strategically helped balance computational resources effectively.

The data collection phase involved regular sampling of PV system parameters, weather data integration, and performance metrics logging. Model training followed with initial training using historical data, establishing a periodic retraining schedule, and validating against performance metrics.

The deployment strategy included a staged rollout to test performance, integration with monitoring systems, and implementation of fallback mechanisms for reliability. Maintenance procedures were equally crucial, encompassing regular model evaluation, performance tracking, update scheduling, and comprehensive error logging and analysis.

System integration tied all these elements together, creating a cohesive solution that addressed the main concerns with providing practical functionality for real-world PV systems. This integrated approach effectively balanced performance requirements with computational efficiency and incorporating robust monitoring mechanisms to ensure reliable operation.

#### Dataset statistic of input and output variables

Table 1 provides detailed descriptions of the datasets and crucial information utilized for model creation. Before the modeling process, both input and output parameters underwent normalization, ensuring they fell within the range of − 1 to 1 as per Eq. (24).

24 $$y_{norm,i} = 2\left( {\frac{{y_{i} - y_{min} }}{{y_{max} - y_{min} }}} \right) - 1$$

where, the normalized value of datapoint i, represented as $y_{norm,i}$, reflects the normalization process, while $y_{i}$ denotes the original value of datapoint i. $y_{min}$ and $y_{max}$ signify the minimum and maximum values across all data points, respectively.

**Table 1:** Dataset statistic of input and output variables.

	Irradiance	Temperature	I_m_	V_m_	P_m_
Count	876,011	876,011	876,011	876,011	876,011
Mean	714.46	29.79	226.85	267.56	61012.82
Std	226.90	5.00	95.93	28.12	26794.11
Min	400.00	25.00	0.00	− 74.46	− 12787.95
25%	600.00	25.00	147.41	271.73	40198.47
50%	800.00	25.00	222.44	272.62	60821.47
75%	1000.00	35.00	369.34	272.97	100316.44
max	1000.00	35.00	372.38	293.18	100660.26

The dataset was split into two groups, with an 80:20 ratio for training, validation, and testing purposes. The training data was primarily utilized for data-driven modeling, while the testing set was employed to evaluate the models’ generalizability. Subsequently, the training set was fed into the models (LR, RR, Lasso R, BR, DTR, GBR, and ANN), and a trial-and-error process was employed to determine the most suitable model architecture.

Upon analyzing Table 1, it is observed that the irradiance column exhibits both mean and median values, with a minimal difference between them, suggesting a standard distribution. In contrast, the temperature and Im columns display a scenario where the mean value exceeds the median, indicating a left-skewed distribution. Conversely, in the Vm and Pm columns, the median value surpasses the mean, signifying a right-skewed distribution.

##### Normalized root mean squared error (NRMSE) metric

We have now conducted an Analysis of Variance (ANOVA) test to assess the statistical significance of the differences in RMSE values across our seven models LR, RR, Lasso R, DTR, BRR, GBR, and ANNR for the three datasets I_m_, V_m_, and P_m_. The ANOVA test is appropriate in this context as it allows us to compare the means of multiple groups (i.e., the RMSE values of different models) and determine if the observed differences are statistically significant. Our analysis yielded an F-statistic of 0.3306 and a *p* value of 0.9097. Since the *p* value is substantially greater than the conventional significance level of 0.05, we conclude that there are no statistically significant differences in RMSE values among the models across the different datasets. This suggests that while the models may exhibit variations in predictive performance, these variations are likely due to chance rather than fundamental differences in the models’ abilities. While this result may seem counterintuitive given the observed differences in RMSE, it is important to note that statistical significance is influenced by several factors, including sample size and variability within the groups. In our case, the large sample size (over 850,000 data points) may have contributed to the high *p* value, as even small differences in means can be statistically significant with large samples. Additionally, the variability in RMSE values across the different datasets and models may have also played a role. We believe that including this statistical analysis provides a more complete picture of our results and strengthens the rigor of our study.

**Fig. 15:**
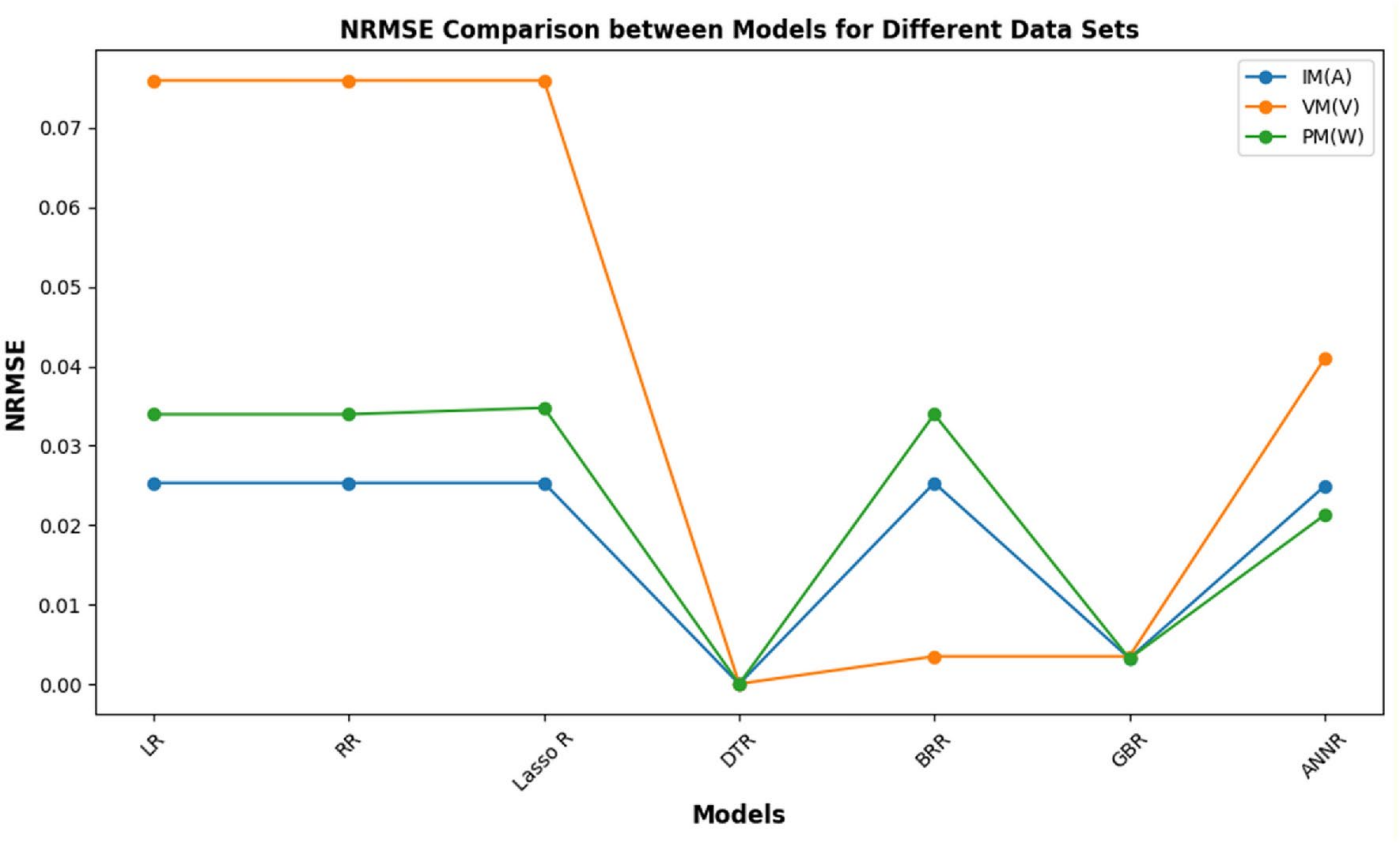
NRMSE comparison between machine learning models.

Key observations from Fig. 15 include the dominance of the DTR, exhibiting exceptionally low NRMSE values across all datasets, nearing zero and indicating its strong predictive performance. The GBR also demonstrates consistently low NRMSE values, highlighting its robustness. The LR, RR, and Lasso Regression models show similar NRMSE values, suggesting comparable performance. The ANNR shows some variability, indicating potential sensitivity to dataset characteristics. Specifically, for I_m_(A), DTR achieves an NRMSE of approximately 1.7e−05, while GBR achieves around 0.0032. For V_m_(V), DTR has an NRMSE of about 4.3e−05, and GBR approximately 0.0035. For P_m_(W), DTR achieves roughly 2.1e−05, and GBR around 0.0032. These values, as depicted in Fig. 18, provide a quantitative basis for comparing model performance. The inclusion of the NRMSE metric, both in the text and Fig. 18, significantly enhances our analysis, providing a more nuanced understanding of model performance, allowing for robust cross-scale comparison, and facilitating a more informed interpretation of the results.

#### Feature correlation analysis

**Fig. 16:**
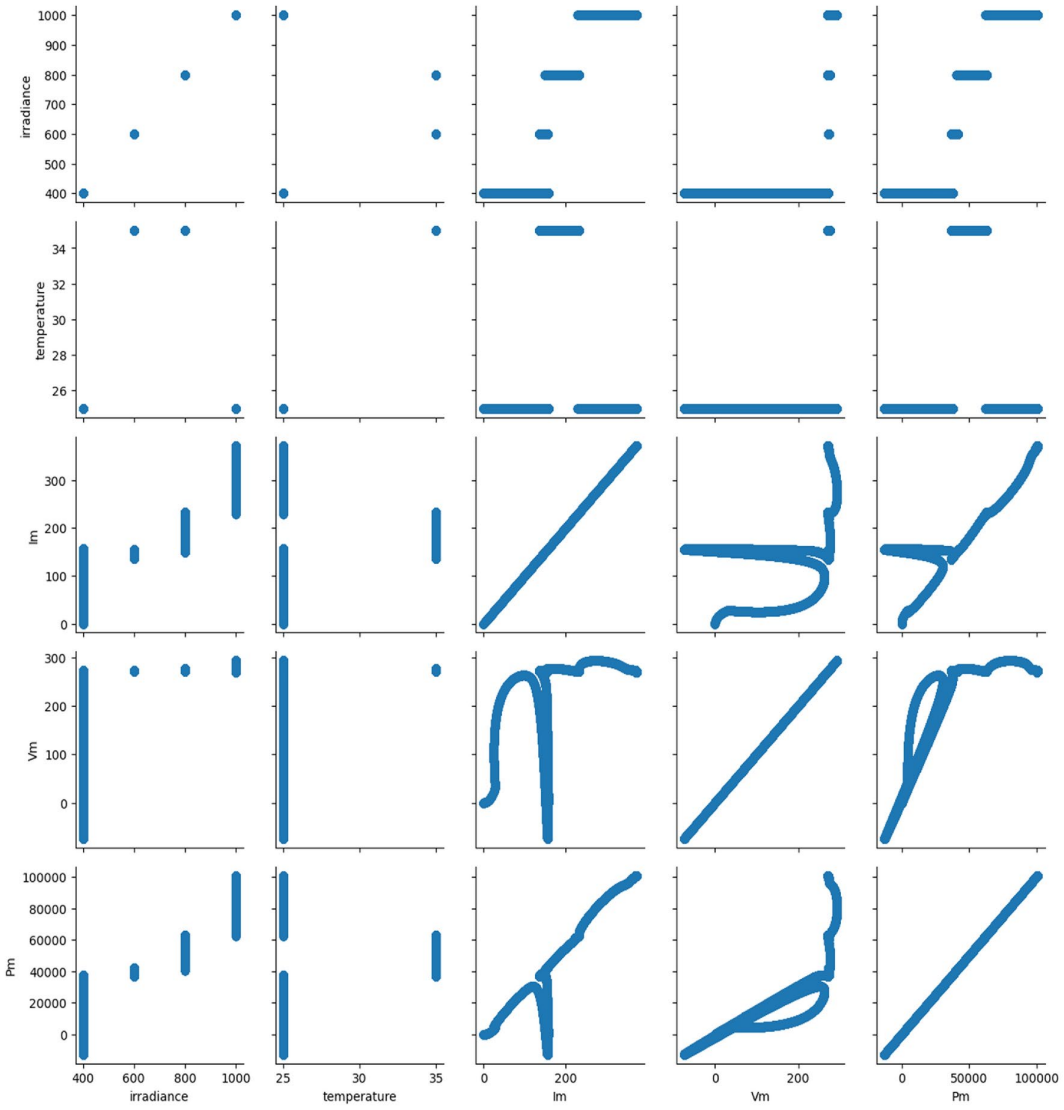
Pairwise relationship of the variables.

**Fig. 17:**
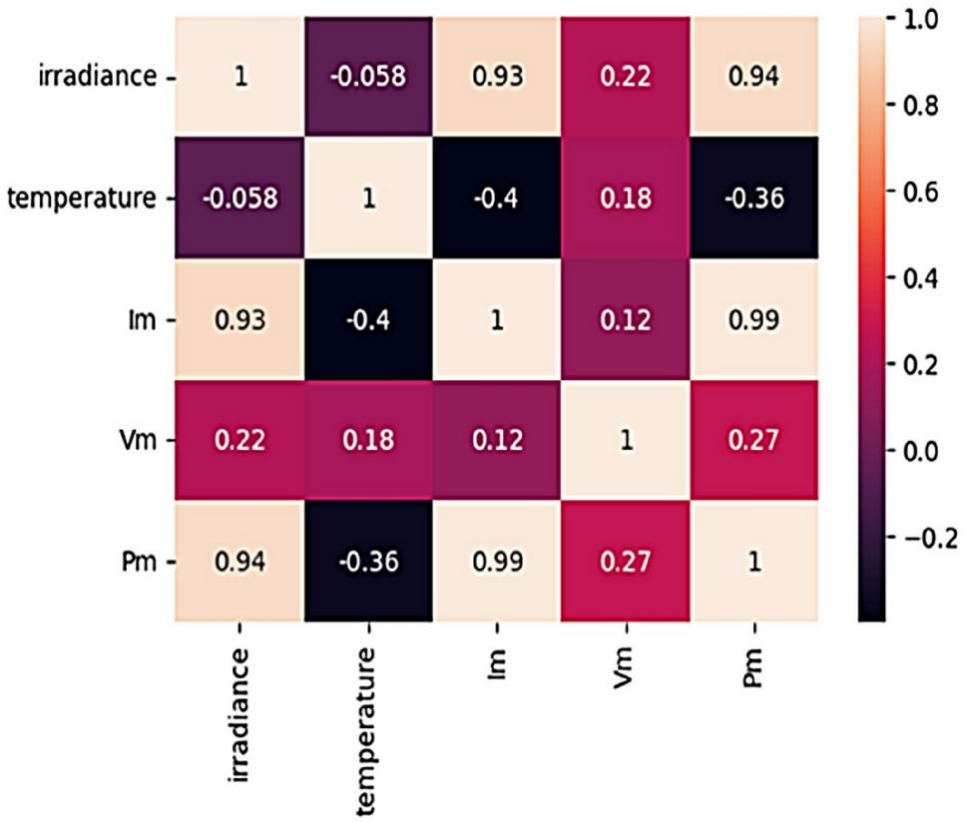
Heatmap of the Pearson correlation parameter among all features, where the color intensity represents the strength of the correlation.

**Fig. 18:**
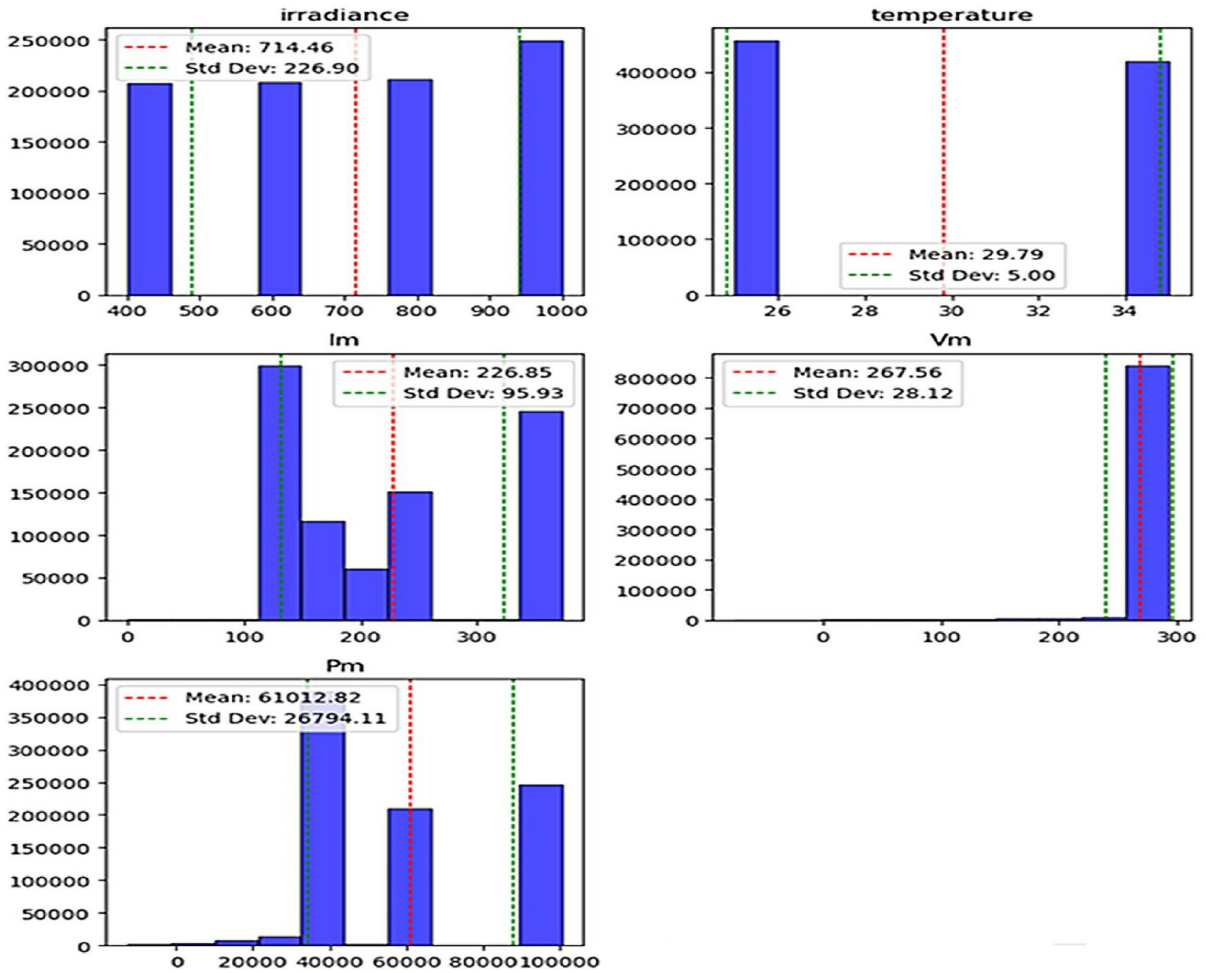
Histogram of normal distribution curve and intensity distribution of output and input features.

Conducting data correlation analysis enhances our understanding of the relationships among various datasets, enabling more effective data analysis, extraction of valuable insights, and practical application of findings. Additionally, it facilitates the identification of potential patterns within the data, which aids in predicting future trends more accurately. In this study, we examined pairwise associations among the data, as depicted in Fig. 16. Pearson correlation is a widely recognized approach for assessing data correlations, providing a measure of the linear relationship between two variables as shown in Fig. 17. The correlation parameter ranges from − 1 to 1, where 1 indicates a perfect positive correlation, 0 indicates there is no linear relationship among variables, and − 1 indicates a perfect negative correlation. It’s noted that irradiance emerges as the most influential factor affecting MPPT, as shown by the Pearson correlation analysis in Fig. 18, consistent with theoretical expectations. The strong correlation between maximum power and irradiance further reinforces this observation, aligning with theoretical principles. The deep learning model utilizes the power, current, and voltage measurements of the PV system as input information and generates a prediction for the input illuminance. Moreover, the correlation is inversely proportional to the darkness of the color, with darker shades (color closer to black) indicating lower correlation, while lighter shades (color closer to white) indicate higher correlation.

The relationship between temperature, irradiance, and photovoltaic (PV) output is inherently nonlinear. PV output generally increases with irradiance, but the effect of temperature is more complex. Higher temperatures tend to reduce the efficiency of PV modules due to a decrease in the open-circuit voltage, which results in lower power output. Therefore, the combined effect of these two variables on PV performance depends on their interaction and the specific material properties of the PV cells. Understanding this nonlinear relationship is crucial for accurate performance modeling and optimization of PV systems under varying environmental conditions. Here’s a more detailed explanation:

*Irradiance and PV Output* PV output is strongly influenced by solar irradiance, which is the amount of sunlight energy reaching the PV surface. Generally, as irradiance increases, the output power of the PV module increases. This is because more photons are available to excite electrons in the semiconductor material, generating more electric current.

*Nonlinear Characteristics* The increase in power output with irradiance is not perfectly linear. Factors such as partial shading, spectral variations in sunlight, and non-uniform irradiance on the module surface can introduce nonlinearity.

*Temperature and PV Output* temperature affects the efficiency of PV modules, typically in a negative way. As the temperature of the PV cells increases, the open-circuit voltage (V_oc_) decreases due to reduced bandgap energy in the semiconductor material. While the short-circuit current (I_sc_) may increase slightly with temperature, this increase is much smaller than the loss in voltage, leading to an overall reduction in power output.

*Interdependence* Temperature and irradiance are not independent; they often vary together in real-world conditions. For example, high irradiance typically leads to increased cell temperatures, intensifying thermal losses and reducing efficiency.

The maximum power point (MPP) of a PV system shifts dynamically due to the combined effects of temperature and irradiance. Maximum power point tracking (MPPT) algorithms are designed to continuously adjust operating voltage and current to optimize power output under these changing conditions.

The proposed method involved collecting data on PV panel performance using PV panel parameters, as outlined in “Case study” section. To assess the tracking accuracy of the RT under different temperature and irradiance conditions, simulations were conducted in intervals of 0.2 s, totaling 0.8 s. During each interval, either the irradiance or temperature was varied while the other remained constant. The variations are summarized in Table 2. Table 2 presents the RT model’s anticipated values for Vm and Im, along with the calculated duty cycle during steady state. Additionally, Figs. 23, 24, and 25 illustrate the I, V, and P waveforms of the solar panel, respectively, using the RT models.

**Table 2:** Values predicted by RT models to calculate D.

Time (s)	$I_{r} \left( {{\text{W}}/{\text{m}}^{2} } \right)$	$T (C^{o} )$	$I_{m} \left( {\text{A}} \right)$	$V_{m} \left( {\text{V}} \right)$	$D$
0 to 0.2	400	25	139.290	272.135	0.1158
0.2 to 0.4	600	35	148.836	271.560	0.1457
0.4 to 0.6	800	35	227.562	271.854	0.3087
0.6 to 0.75	1000	25	370.143	272.210	0.4576

#### Histogram distribution

The histogram distribution in Fig. 18 illustrates the features used in developing the ML models, along with their corresponding mean and standard deviation (STD) values. Examining the distribution curves provides several notes on the dataset. As depicted in Fig. 12, the V_m_ distribution appears left-skewed, with a mean of 267.56 and a standard deviation of 28.12. Significantly, the Pm data is predominantly clustered around the mean value of 61,012.82, with a standard deviation of 26794.11. Similarly, for I_m_, most of the data is centered around the mean value of 226.85, with a standard deviation of 95.93. Additionally, the temperature data exhibits a Bernoulli distribution, with the majority of data centered around the mean value of 29.79 and a standard deviation of 5.0. Finally, regarding irradiance, the bulk of the data is concentrated around the mean value of 714.46, with a standard deviation of 226.90.

### Sample size effect

**Fig. 19:**
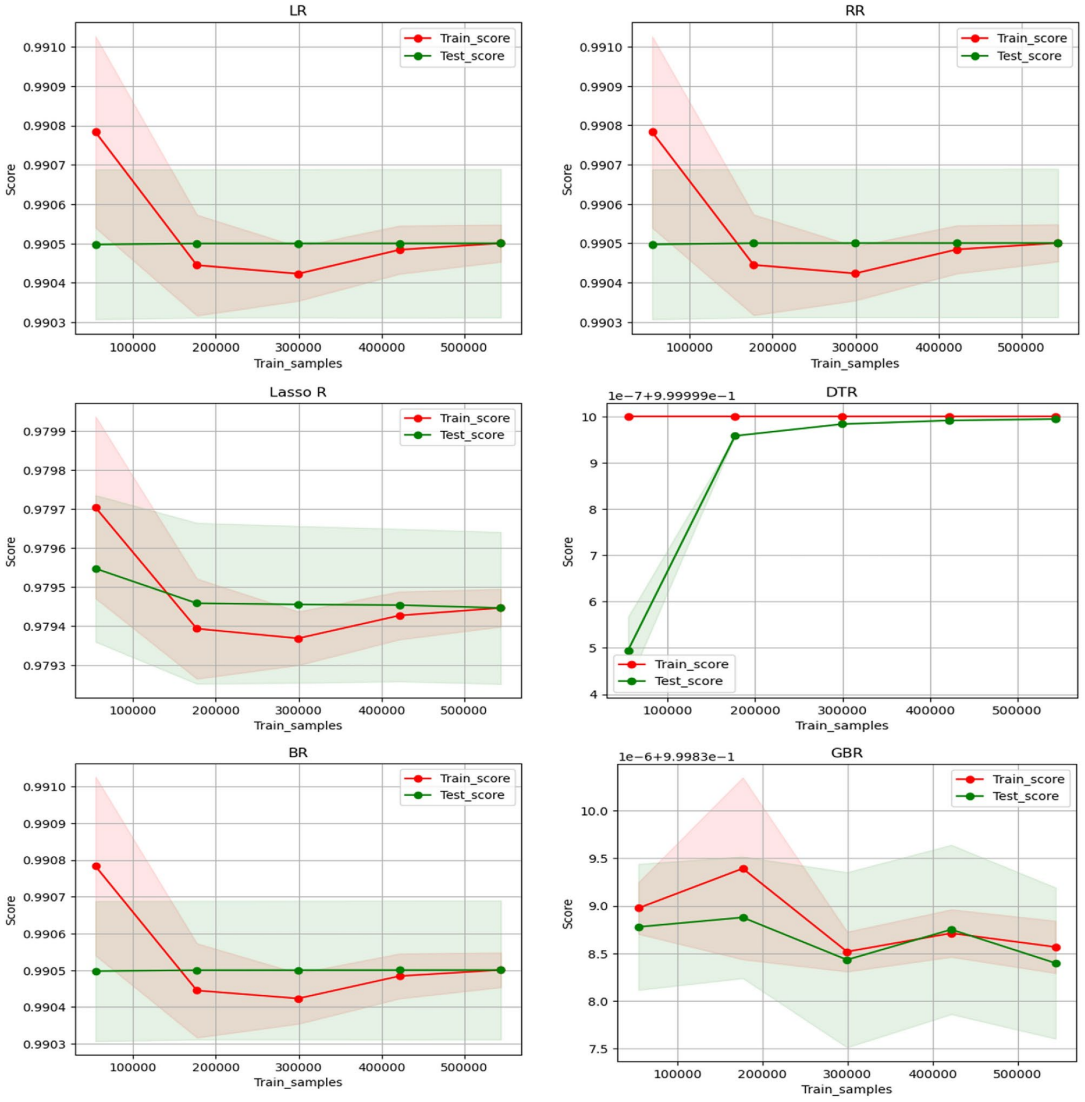
Comparison of R^2^ data on testing and training set for I_m_.

Data-intensive training is often required to achieve excellent results in machine learning. However, the exact amount of data desired is determined by evaluating its actual effect on model effectiveness. This evaluation is performed by examining the ${\text{R}}^{2}$ values of the model on all the testing and training datasets. The model’s ability to generalize among the two groups is assessed based on smaller ${\text{R}}^{2}$ errors, which indicate stronger generalization capabilities. The dataset underwent random division into five partitions to aid in model training. Figure 19 shows the various machine learning models for ${\text{R}}^{2}$ comparisons in both the test and training sets to predict Im. It is observed that as the sample size increases, the ${\text{R}}^{2}$ value of the model also rises. The DTR model outperforms other models in the test set in terms of ${\text{R}}^{2}$ when the sample size is less than 200,000, demonstrating superior performance with lower sample sizes. Regarding model generalization, almost identical ${\text{R}}^{2}$ data appear among the testing and training sets when the sample size reaches 400,850 for the LR, RR, BR, and Lasso R models. Although with relatively smaller ${\text{R}}^{2}$ scores, this indicates strong generalization abilities.

**Fig. 20:**
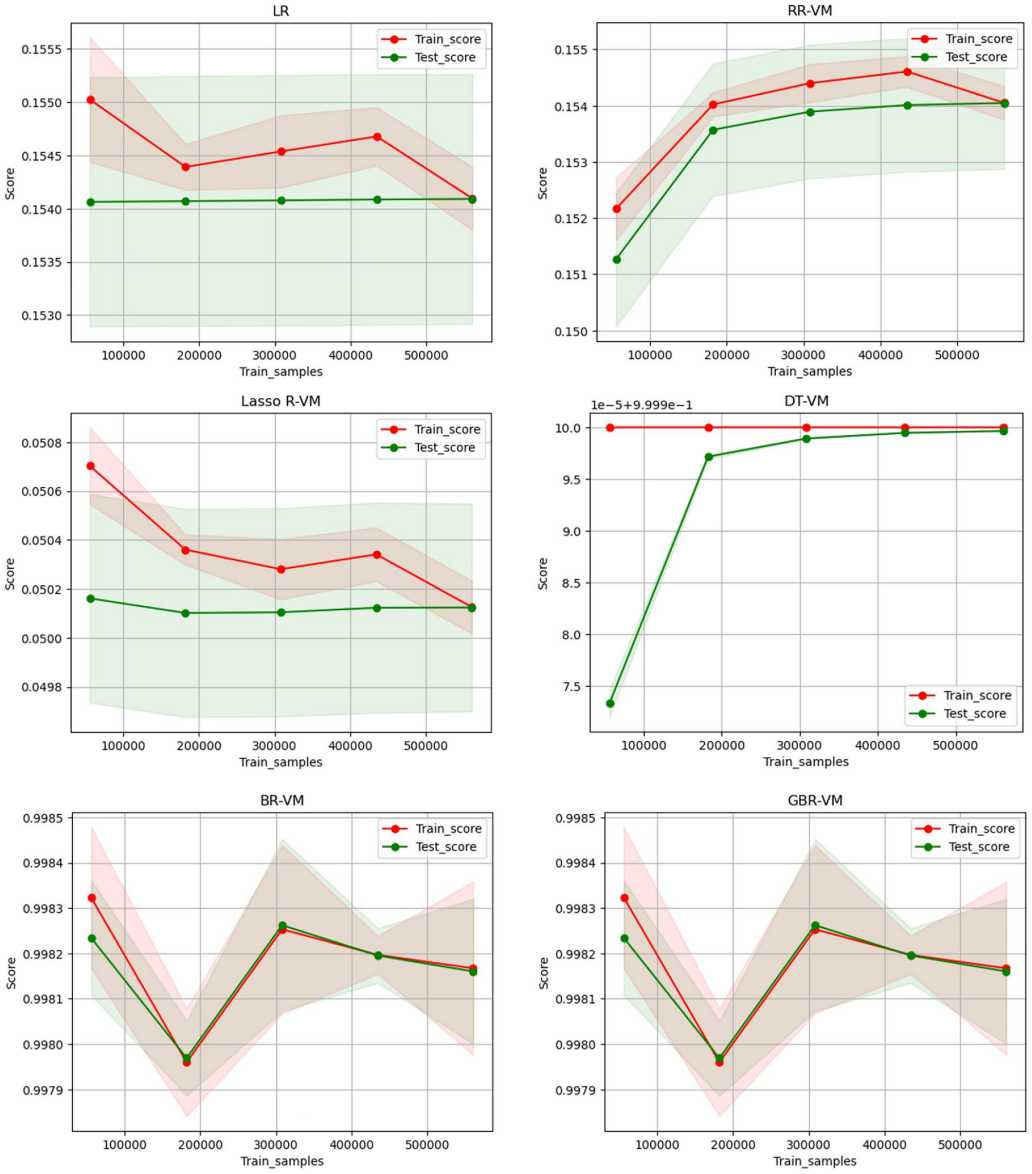
Comparison of R^2^ data on testing and training set for V_m_.

Figure 20 illustrates the comparison of ${\text{R}}^{2}$ values among different machine learning models on all testing and training sets for predicting V_m_. As the sample size increases, the model’s ${\text{R}}^{2}$ value demonstrates a corresponding increase, as illustrated Fig. 20. For sample sizes below 200,000, the DTR model consistently shows superior effectiveness in terms of ${\text{R}}^{2}$ on the test set when compared to the other models. This highlights the superior training performance of the DTR model when dealing with lower sample sizes. Additionally, both the BR and GBR models exhibit similar training and test scores when the sample size is below 200,000. However, they show some fluctuations beyond that point, although the ${\text{R}}^{2}$ values remain relatively high for both cases.

**Fig. 21:**
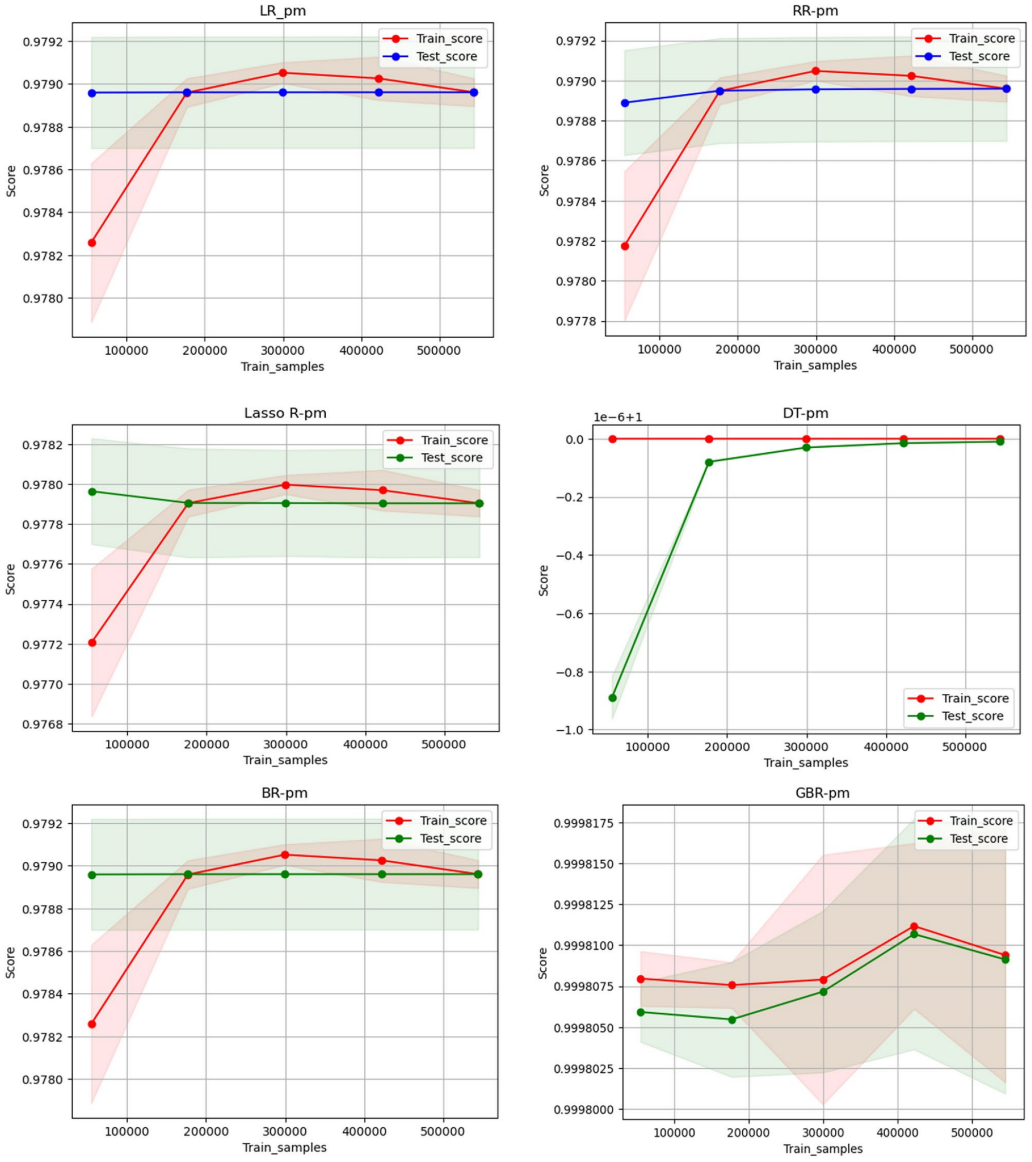
Comparison of R^2^ data on testing and training set for P_m_.

Figure 21 illustrates the comparison of ${\text{R}}^{2}$ values among different machine learning models on all testing and training sets for predicting P_m_. As the sample size increases, there is a noticeable rise in the ${\text{R}}^{2}$ value of the model, as illustrated in Fig. 21. For sample sizes less than 200,000, the DTR model consistently shows better performance. This highlights the superior training performance of the DTR model when dealing with lower sample sizes. The LR, RR, BR, and Lasso R models show almost identical ${\text{R}}^{2}$ values among the testing and training sets when the sample size reaches 500,850, indicating strong generalization abilities despite the relatively lower ${\text{R}}^{2}$ score. Furthermore, the GBR model exhibits a notably high score, peaking at a sample size of 400,200, before experiencing a slight decrease.

In this study, we employed a comprehensive approach to error analysis and outlier management to ensure the robustness and reliability of our machine learning models. With a substantial dataset comprising 876,012 data points, we prioritized rigorous data preprocessing techniques. Initially, we addressed data redundancy by identifying and removing duplicate entries using the duplicated function in Python, resulting in the elimination of 25,219 instances. This step ensured data integrity and prevented potential bias in model training. Furthermore, we thoroughly investigated the presence of outliers within our dataset. While no distinct outliers were identified, we implemented robust error analysis measures to evaluate the performance of our seven machine learning models LR, RR, Lasso R, BR, DTR, GBR, and ANNacross the three data types IM, VM, and PM. Specifically, we calculated the RMSE, MAE, and R^2^ as key metrics to assess the accuracy and goodness of fit of our models. These metrics provided a comprehensive evaluation of the predictive capabilities of each model, considering both the magnitude and direction of errors.Moreover, we acknowledge that the absence of explicit outlier identification does not preclude the possibility of localized or subtle anomalies within the data. To mitigate the potential impact of such anomalies on model performance, we employed techniques such as regularization (Lasso R) and ensemble methods (GBR) that are inherently less sensitive to outliers. Additionally, we conducted sensitivity analyses by systematically varying model parameters and hyperparameters to assess the stability and consistency of our results. However, it is important to note that while the preprocessing steps ensured data quality, a more detailed error analysis could provide further insights into the models performance. This could include an examination of residuals to identify any patterns or biases that were not apparent in the initial analysis. Such an analysis would enhance the understanding of the models’ predictive capabilities and their limitations, offering a more comprehensive evaluation of their effectiveness. By delving deeper into the residuals, we could uncover any systematic errors or trends that might have been overlooked, thereby refining the models and improving their accuracy. This additional layer of analysis would not only validate the robustness of our models but also provide a clearer picture of their practical applicability in real-world scenarios.

### Evaluation of the performance of ML models

**Fig. 22:**
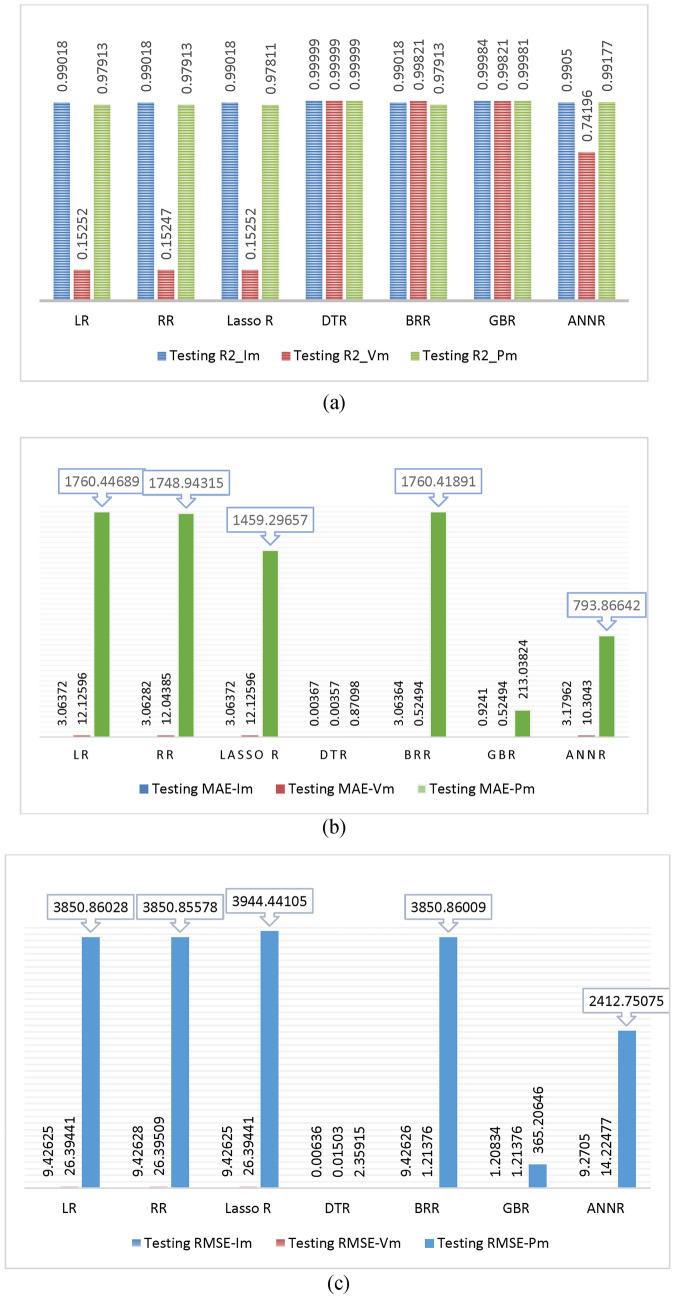
Bar chart illustrating the testing evaluation using (**a**) R^2^, (**b**) MAE, and (**c**) RMSE as evaluation metrics for all ML models.

Evaluation metrics such as R^2^, MAE, and RMSE are widely used to assess the effectiveness of machine learning (ML) models to a certain degree. In this study, seven ML algorithms—LR, RR, Lasso R, BR, DTR, GBR, and ANN—were evaluated through a comparative analysis. Figure 16 illustrates the performance of these algorithms on testing data. As seen in Fig. 22a, the testing performance is generally favorable, with R^2^ values exceeding 0.9901, 0.15, and 0.97 for predicting maximum current, voltage, and power, respectively. DTR delivers the most accurate predictions, achieving an R^2^ value of 0.99999. While testing times are fairly similar, ANN exhibits a slightly longer testing time of 1.059 s. In Fig. 22b, the MAE values across all algorithms remain low, below 0.00389, 0.00457, and 0.97 for predicting maximum current, voltage, and power, respectively. Again, DTR outperforms other models, yielding MAE values of 0.00367, 0.00357, and 0.87098 for these parameters. Similarly, Fig. 22c demonstrates that the RMSE values are below 0.00746, 0.02514, and 2.66 for predicting maximum current, voltage, and power, respectively, with DTR emerging as the top performer, achieving RMSE values of 0.00636, 0.01503, and 2.35915, respectively. The superior performance of DTR can be attributed to several factors. First, its ability to model non-linear relationships between features and target variables plays a crucial role. Unlike linear regression, which assumes linearity, decision trees can capture complex patterns in the data without requiring such assumptions. This flexibility allows DTR to adapt effectively to diverse data distributions and relationships. Additionally, decision trees are relatively simple to implement and use compared to some other ML models, making them a practical choice for various tasks.

**Fig. 23:**
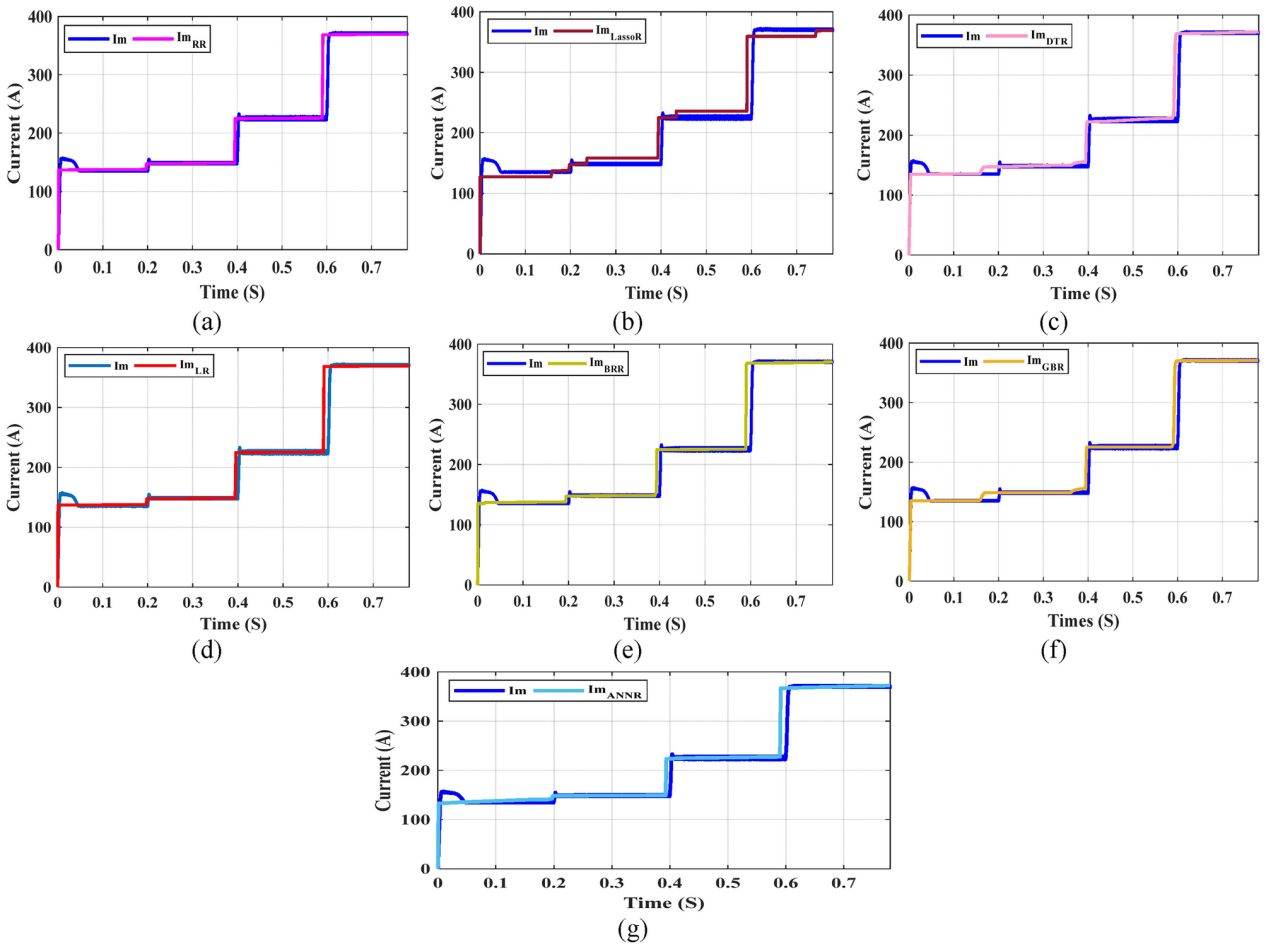
Comparison of the real Im with Im predicted by ML models: (**a**) LR, (**b**) RR, (**c**) Lasso R, (**d**) DTR, (**e**) BR, (**f**) GBR, and (**g**) ANN.

**Fig. 24:**
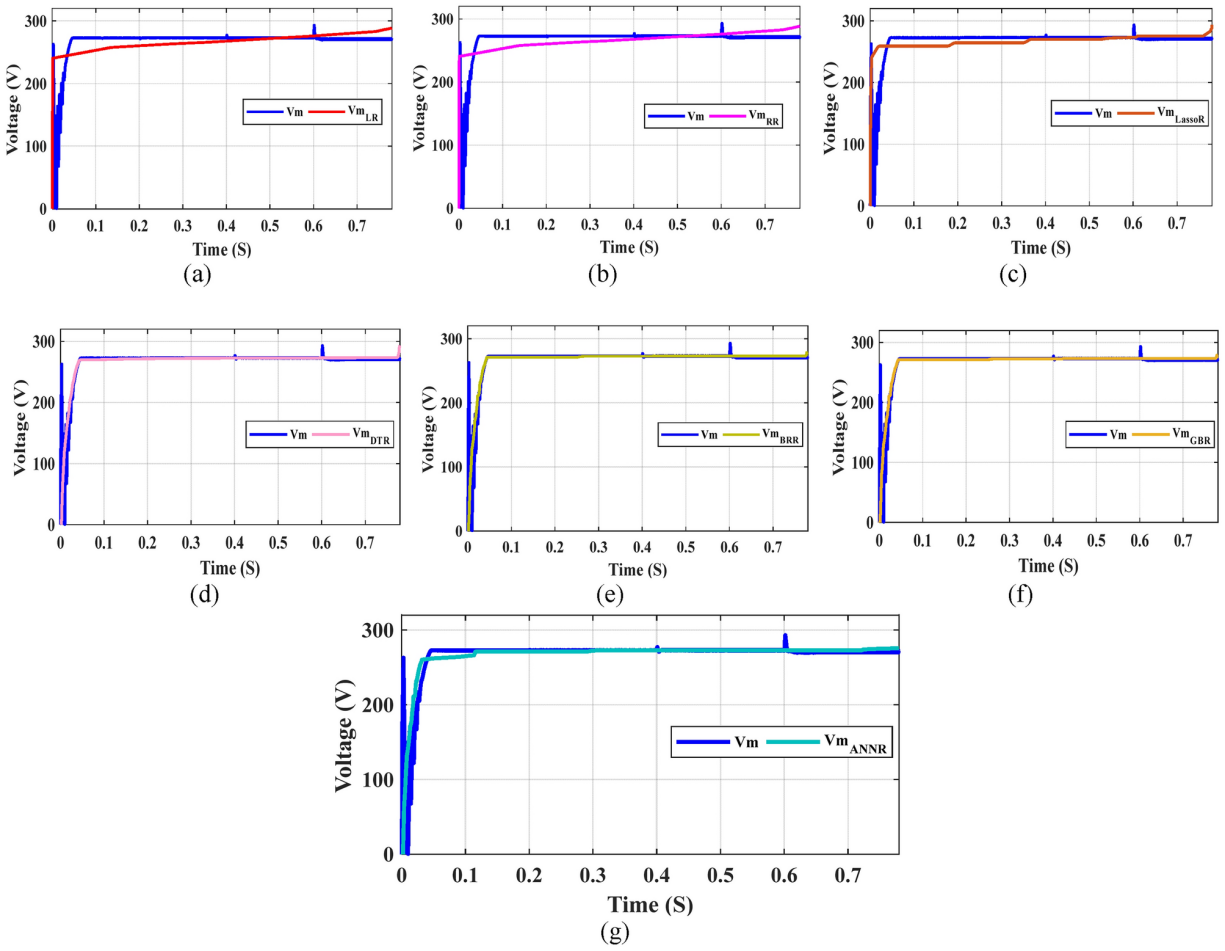
Comparison of the real Vm values in the test dataset with the Vm values predicted by various ML models: (**a**) LR, (**b**) RR, (**c**) Lasso R, (**d**) DTR, (**e**) BR, (**f**) GBR and (**g**) ANN.

**Fig. 25:**
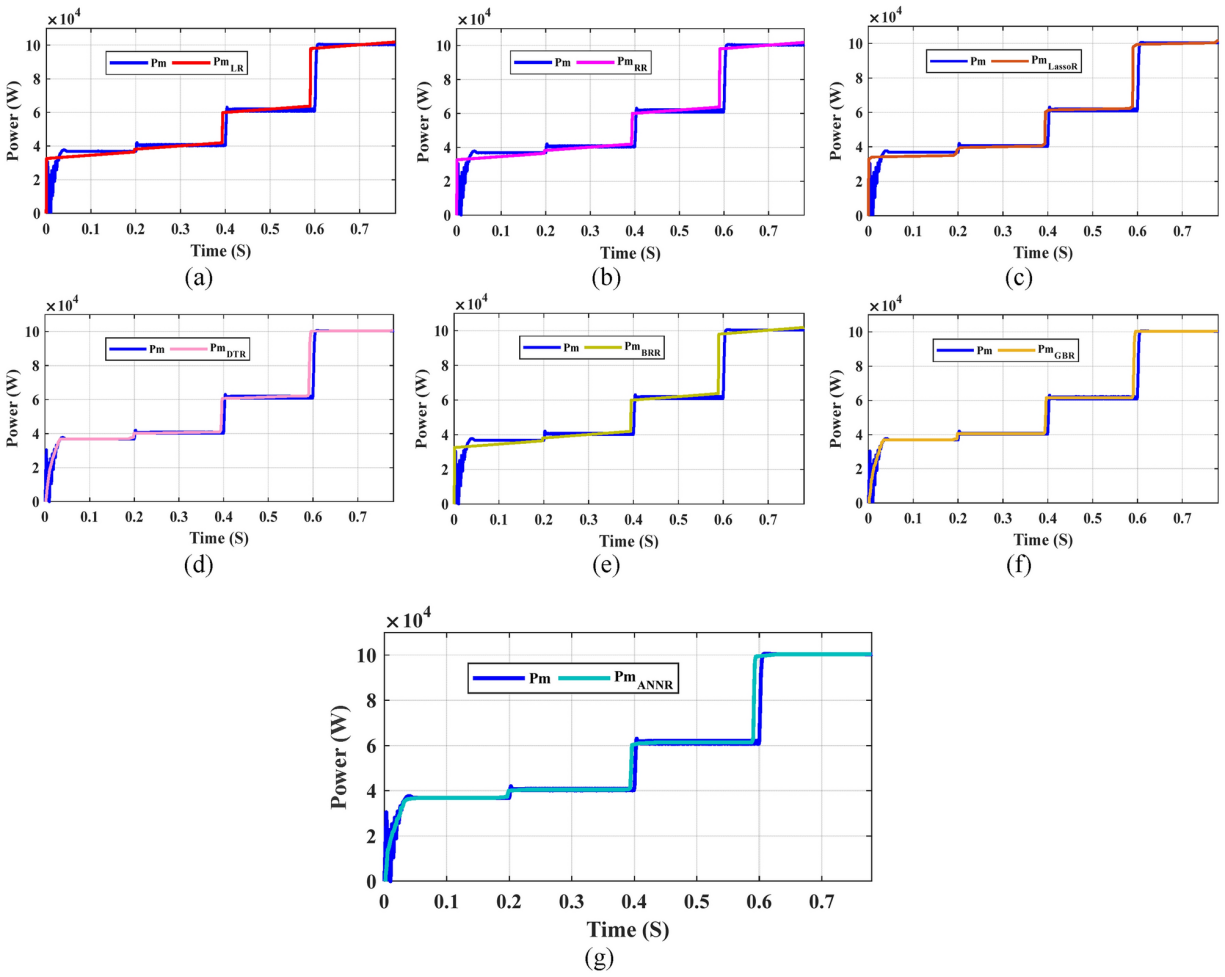
Comparison of the real Pm with Pm predicted by ML models: (**a**) LR, (**b**) RR, (**c**) Lasso R, (**d**) DTR, (**e**) BR, (**f**) GBR and (**g**) ANN.

Furthermore, Figs. 23, 24, and 25 present a comparison between the predicted tracking MPP values and the true values for various test samples. These figures unequivocally demonstrate the close alignment between the predicted I_m_, V_m_, and P_m_ values and the true values. This observation solidifies the DTR as the machine learning algorithm with the lowest prediction error among the models used in this study. The complexity of the dataset poses challenges for other machine learning models, which fail to achieve better results than a simple feature discordance calculation. Consequently, the DTR model emerges as the preferred choice in this study. However, it is important to acknowledge a weakness inherent in the DTR model, which is the time required for training and fitting. Training DTR techniques typically take longer compared to traditional machine learning algorithms due to their nature. Despite this, the testing time of the model remains relatively short in comparison to the training time. Additionally, a high RMSE value is undesirable, emphasizing the need for further improvement in tracking MPP prediction using such systems. The ultimate objective is to develop a successful machine learning application and integrate the model into expert systems. Experimental findings reveal that the DTR model achieves approximately 0.006 for I_m_, 0.015 for V_m_, and 2.36 for P_m_ in terms of prediction performance, outperforming other models in tracking MPP prediction. These results highlight the potential for deploying an ensemble-based intelligent expert system with an integrated DTR model, which can alleviate pressure and save professional time in this field. Such a system would autonomously predict tracking MPP using the provided inputs for the ensemble model in this study.

## Conclusion

This study delved into the examination of seven distinct machine learning (ML) models, LR, RR, Lasso R, BR, DTR, GBR, and ANN, to predict parameters, namely I_m_, V_m_, and MPP, within a Standalone photovoltaic (PV) system. The input variables selected for this analysis were irradiance and temperature, which were gleaned from experimental data collected from the Standalone PV system. Through a comprehensive evaluation process utilizing metrics such as MAE, ${\text{R}}^{2}$ values, and RMSE, the DTR model emerged as the preeminent performer across all parameters examined. This comprehensive assessment allowed for a nuanced understanding of each model’s predictive capabilities and their efficacy in capturing the complex relationships within the dataset. The hierarchical ranking of ML models’ performance revealed insights into their relative strengths and weaknesses in predicting I_m_, V_m_, and P_m_. For I_m_, the DTR model exhibited the highest performance, followed by GBR, ANN, LR, Lasso R, BR, and RR. Similarly, for V_m_, the DTR model led the rankings, succeeded by BR, GBR, ANN, LR, Lasso R, and RR. Lastly, for P_m_, the DTR model demonstrated superior performance, outperforming GBR, ANN, RR, LR, BR, and Lasso R.

**Fig. 26:**
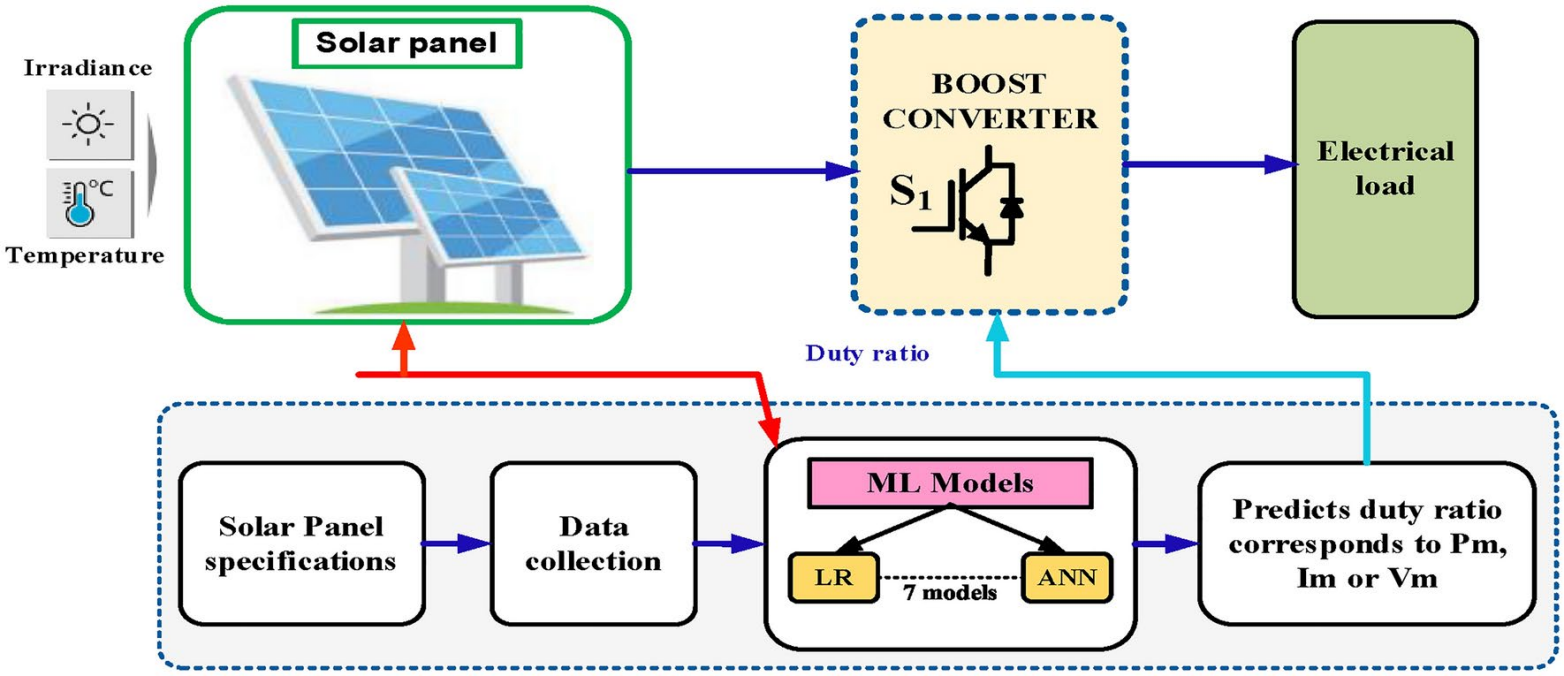
solar power system utilizing machine learning models.

The Fig. 26, illustrates an intelligent solar power system that combines traditional hardware components with modern machine learning techniques. At its core, the system starts with a solar panel that converts solar energy into electrical power. This power then flows through a boost converter (marked as S1), which is an essential power electronics device that steps up the voltage to a level suitable for the electrical load. The electrical load represents the end-user consumption point, which could be anything from household appliances to industrial equipment. The system’s performance is influenced by environmental factors, specifically temperature and irradiance (solar radiation intensity), which are monitored and fed into the control system.

What makes this system particularly sophisticated is its integration of machine learning models for optimization. The bottom portion of the diagram shows the analytical framework: it begins with collecting solar panel specifications and operational data, which are then processed through two different machine learning models—Linear Regression (LR) and Artificial Neural Network (ANN). These models analyze the collected data to predict the optimal duty ratio correction for the input voltage (V_in_). The duty ratio is a critical parameter that controls how the boost converter operates, directly affecting the system’s efficiency. The system implements a feedback mechanism, shown by the red and blue lines, where the predicted duty ratio is fed back to the boost converter, creating a continuous optimization loop. This machine learning-enhanced approach allows the system to adapt to changing environmental conditions and maintain optimal performance throughout its operation.

## Data Availability

All data generated or analyzed during this study are included in this published article. You can contact Dr. Mohamed A. Ghalib in case of requesting study data. this email: Mohamed01177@techedu.bsu.edu.eg.
